# Exploratory Integration of Metabolomics and Machine Learning Identifies Candidate Signatures of Spontaneous Preterm Birth

**DOI:** 10.3390/biomedicines14102188

**Published:** 2026-09-27

**Authors:** Maicol Avellaneda-Arciniegas, Carlos H. Becerra-Mojica, Mónica P. Cala, Bladimiro Rincón-Orozco, William Hidalgo

**Affiliations:** 1 School of Medicine, Universidad Industrial de Santander, Bucaramanga 680002, Colombia; maicol.avellaneda@correo.uis.edu.co (M.A.-A.); cbecerra@uis.edu.co (C.H.B.-M.); blrincon@uis.edu.co (B.R.-O.); 2 MetCore—Metabolomics Core Facility, Vice-Presidency for Research, Universidad de los Andes, Bogota 111711, Colombia; mp.cala10@uniandes.edu.co; 3 School of Chemistry, Universidad Industrial de Santander, Bucaramanga 680002, Colombia

**Keywords:** spontaneous preterm birth, first trimester, metabolomic, machine learning, plasma, lipids

## Abstract

**Background/Objectives:** Spontaneous preterm birth (sPTB) remains a major cause of neonatal morbidity and mortality. This study aimed to characterize first-trimester plasma metabolic alterations associated with sPTB and evaluate their discriminatory potential using metabolomics and machine learning. **Methods:** This retrospective case–control study included 199 pregnant women (64 sPTB and 135 term births) from the Colombian Cohort for the Early Prediction of Preterm Birth (COLPRET). First-trimester plasma was analyzed by Liquid Chromatography–Quadrupole Time-of-Flight Mass Spectrometry (LC-QTOF-MS). Multivariate and univariate analyses were used to characterize metabolic differences. Thirty candidate metabolites were selected through outcome-associated univariate analyses performed on the complete cohort before subsequent machine-learning model evaluation. Six machine-learning algorithms were evaluated using clinical, metabolomic, and integrated predictor sets within a nested stratified cross-validation framework. **Results:** Lipid-related metabolites, particularly lysophospholipids and phospholipids, predominated among the molecular alterations associated with sPTB. Lysophosphatidylcholine O-14:0 (LPC O-14:0) showed the highest individual discrimination with an area under the receiver operating characteristic curve (ROC-AUC) (AUC = 0.705). Models based on clinical data showed limited discrimination (out-of-fold [OOF] ROC–AUC: 0.500–0.555), whereas metabolomic and integrated models achieved OOF ROC–AUC values of 0.632–0.760 and 0.641–0.770, respectively. Metabolomic and integrated models generally showed higher ROC–AUC estimates than clinical models. However, adding the five clinical variables to the metabolomic predictor set did not significantly improve the ROC–AUC after correction for multiple comparisons (all false discovery rate [FDR]-adjusted q ≥ 0.060). **Conclusions:** First-trimester plasma metabolomics identified predominantly lipid-related alterations associated with subsequent sPTB. Metabolomic information showed greater discriminatory capacity than the five evaluated clinical variables, with no robust evidence of additional discrimination from their integration. These exploratory findings require validation in larger prospective cohorts before clinical utility can be established.

## 1. Introduction

Preterm birth (PTB), defined as delivery before 37 weeks of gestation, remains a major global cause of neonatal morbidity and mortality, accounting for nearly one million deaths annually among children under five years of age [1,2]. The consequences of prematurity, however, extend beyond the neonatal period and may adversely affect child health and development. Children born preterm are at increased risk of impaired psychomotor and neurodevelopmental outcomes, including cognitive, neurological, and sensory difficulties [3]. These effects may persist into later life, with evidence indicating long-term consequences for cognitive functioning and mental health even among individuals born moderately or late preterm [4]. Together, these short- and long-term consequences highlight the clinical importance for identifying pregnancies at increased risk of PTB as early as possible and support the investigation of early biological markers associated with subsequent preterm delivery.

Despite its global burden, the underlying mechanisms triggering PTB are not fully understood, reflecting its multifactorial nature involving the inflammatory, endocrine, infectious, and metabolic pathways [5,6]. This complexity has hindered the development of reliable tools for early prediction, particularly during the first trimester of pregnancy.

Current predictive approaches rely on a combination of clinical risk factors, ultrasound measurements, and biochemical markers such as fetal fibronectin, insulin-like growth factor-binding protein-1 (IGFBP-1), and placental alpha-microglobulin-1 (PAMG-1). However, these markers are typically assessed in later stages of pregnancy, limiting their utility for early risk stratification and timely intervention [7,8]. Nevertheless, growing evidence suggests that biological alterations associated with subsequent PTB may already be detectable during the first trimester. Routinely available biochemical markers such as pregnancy-associated plasma protein-A (PAPP-A) and free β-human chorionic gonadotropin (free β-hCG) have been associated with subsequent PTB and spontaneous preterm birth (sPTB), although their standalone discriminatory performance remains limited (area under the receiver operating characteristic curve [ROC-AUC] < 0.70) [9]. Furthermore, recent studies have therefore explored multimarker strategies, including first-trimester serum protein panels combined with machine-learning approaches, further supporting the investigation of complementary biological information rather than reliance on individual biomarkers [10].

Consistent with this growing evidence, recent studies conducted by our research group, the Laboratory of Molecular Genetics of Infectious Diseases and Cancer (GEMEICA), have identified potential first-trimester markers associated with (sPTB). Notably, our previous findings showed that lower plasma concentrations of complement factor H during the first trimester have been significantly associated with subsequent sPTB, supporting its potential as an early biological marker [11]. In addition, the first-trimester cervical consistency index (CCI) has shown potential predictive value for sPTB, particularly for deliveries occurring before 34 weeks of gestation [12]. Consequently, there is a critical need to identify early biomarkers that capture the biological processes preceding the onset of sPTB.

In this context, previous metabolomic studies have identified maternal serum metabolites associated with subsequent sPTB, including lipid-related signals, although their discriminatory performance has generally remained moderate and external validation is still required [13]. These observations support the investigation of early biological markers associated with sPTB. Metabolomics has emerged as a powerful tool for characterizing global metabolic alterations associated with complex diseases and may provide insights into the biological processes underlying spontaneous preterm birth. By enabling the detection of small-molecule metabolites that reflect real-time physiological and pathological processes, metabolomics provides a unique opportunity to uncover early biochemical signatures of sPTB, particularly those linked to inflammation, oxidative stress, and lipid metabolism. Recent advances in high-resolution mass spectrometry and data analysis have further enhanced the potential of metabolomics in biomarker discovery [14,15].

Moreover, the integration of metabolomic data with machine learning (ML) approaches offers a promising framework for evaluating complex molecular patterns in heterogeneous clinical conditions such as sPTB. ML models, including support vector machines (SVMs) and gradient boosting algorithms, can handle high-dimensional datasets and identify patterns that may not be evident through conventional statistical approaches [16]. However, evidence integrating first-trimester metabolomic profiling with ML approaches for sPTB remains limited, particularly in Latin American populations. Therefore, this study aimed to characterize first-trimester plasma metabolomic profiles associated with subsequent sPTB and to explore the discriminatory performance of metabolomic and integrated metabolomic-clinical signatures using machine-learning approaches.

## 2. Materials and Methods

### 2.1. Study Design and Population

Participants were recruited from the Colombian Cohort for the Early Prediction of Preterm Birth (COLPRET), previously described by Becerra-Mojica et al. 2022 [17]. Beginning in January 2020, pregnant women attending first-trimester screening at the maternal-fetal medicine units of Hospital Universitario de Santander and Centro de Atención Materno Fetal INUTERO in Bucaramanga, Colombia, were consecutively invited to participate. Eligible participants had a singleton pregnancy between 11 + 0 and 13 + 6 weeks of gestation and planned to remain in the study area throughout pregnancy and delivery. Women with a history of cancer, human immunodeficiency virus (HIV) infection, or cervical surgery were excluded from the cohort [17].

For the retrospective case–control analysis, eligible COLPRET participants were selected according to pregnancy outcome and the availability of an adequate stored plasma sample for metabolomic analysis. The final analytical sample comprised 199 women, including 64 who subsequently experienced spontaneous preterm birth (sPTB) and 135 who delivered at term (≥37 weeks). sPTB was defined as spontaneous delivery at 37 weeks of gestation without preterm prelabor rupture of membranes (pPROM). Pregnancies complicated by pPROM and preterm deliveries resulting from maternal or fetal medical indications were excluded from the analysis. Thus, all 64 sPTB cases included in the study corresponded to spontaneous preterm births without pPROM [17].

### 2.2. Study Population

Plasma samples were obtained from a larger research project aimed at developing an early prediction strategy for preterm birth through the integration of maternal characteristics, cervical ultrasound measurements, vaginal microbiome composition, and multi-omics profiles in maternal blood. Inclusion criteria comprised women with singleton pregnancies between 11 + 0 and 13 + 6 weeks of gestation, including both nulliparous women and those with a history of sPTB. Exclusion criteria included women with chronic or autoimmune diseases, history of cervical surgery or Müllerian anomalies, medically indicated preterm birth due to maternal or fetal conditions, and pregnancies ending before 22 weeks of gestation [17].

All participants provided written informed consent prior to sample collection. Confidentiality and data protection were ensured in accordance with applicable ethical regulations. Participants were recruited during routine prenatal care visits.

### 2.3. Clinical and Obstetric Statistical Analysis

Clinical and obstetric variables were summarized as median (interquartile range) for continuous variables and frequency (percentage) for categorical variables. Comparisons between women with preterm birth and term birth were performed using the Mann–Whitney U test for continuous variables because data were not normally distributed. Categorical variables were compared using the chi-square test or Fisher’s exact test, as appropriate. Odds ratios (ORs) with 95% confidence intervals (95% CI) were calculated for categorical variables when applicable. A *p*-value < 0.05 was considered statistically significant.

### 2.4. Sample Collection and Metabolomic Analysis

Maternal blood samples were collected using a vacuum technique through venipuncture of the antecubital vein into ethylenediaminetetraacetic acid (EDTA)-containing tubes. Samples were centrifuged at 3000 rpm for 10 min at 4 °C to separate plasma. Plasma aliquots (700 µL) were prepared and stored at −80 °C until further analysis. Metabolite extraction was performed as follows. Plasma samples were thawed on ice for approximately 180 min. A 100 µL aliquot of plasma was then mixed with 300 µL of cold methanol (−20 °C) to induce protein precipitation. The mixture was vortexed for 5 min and subsequently incubated at −20 °C for 20 min. Samples were then centrifuged at 14,000 rpm for 10 min at 4 °C, after which the supernatant was carefully collected and stored at −80 °C until further analysis [18].

Untargeted metabolomic profiling was performed using reverse-phase Liquid Chromatography coupled to a Quadrupole Time-of-Flight Mass Spectrometry (LC-QTOF-MS) Agilent 6545 instrument (Agilent Technologies Inc., Santa Clara, CA, USA) equipped with an electrospray ionization (ESI) source. Chromatographic separation was performed using an InfinityLab Poroshell 120 EC-C18 column (2.1 × 100 mm, 1.9 µm; Agilent Technologies Inc., Santa Clara, CA, USA) maintained at 40 °C and a gradient elution composed of 0.1% (*v*/*v*) formic acid in Milli-Q water (Phase A) and 0.1% (*v*/*v*) formic acid in acetonitrile (Phase B) with a constant flow rate of 0.4 mL/min. The elution gradient started with 5% Phase B, increasing to 95% B during the first 15 min, and was kept constant for 1 min. Finally, the percentage of Phase B was reduced to 5% from minute 16 to 18, remaining at the initial conditions for 5 min for a final analysis time of 22 min. Continuous mass calibration was achieved using reference masses *m*/*z* 121.0509 ($C_{5}H_{4}N_{4})$ and 922.0098 $\left(C_{18}H_{18}O_{6}N_{3}P_{3}F_{24}\right)$ in positive mode (ESI+). Data acquisition was performed in centroid mode over a mass range of *m/z* 100–1100 at a rate of 1.02 spectra/s [18].

### 2.5. Quality Assurance and Quality Control (QA/QC)

To ensure analytical reproducibility and system stability, quality control (QC) samples were prepared by pooling equal aliquots (10 µL) from all study samples. QC samples were used to monitor the consistency of sample preparation and the performance of the analytical platform throughout the acquisition sequence. QC samples were injected at regular intervals (every ten samples) during the analytical run to assess signal stability, reproducibility, and potential batch effects, following established metabolomics quality control practices [19].

### 2.6. Data Processing and Preprocessing

Raw LC-QTOF-MS data were processed using Agilent MassHunter Profinder (version 10.0) for peak deconvolution, alignment, and integration. Additional data processing and feature inspection were performed using Mass Profiler Professional and MassHunter Quantitative Analysis. A total of 853 molecular features were initially detected. Features associated with the extraction blank were subsequently filtered, leaving 642 features for further processing.

Signal drift and systematic analytical variability were corrected using the Systematic Error Removal using Random Forest (SERRF) approach based on quality-control samples [20]. Analytical reproducibility was subsequently assessed using the coefficient of variation (CV) across QC samples, and features with a QC CV ≥20% were excluded, resulting in 560 retained molecular features [21]. A presence filter was then applied, retaining features detected in at least 80% of samples according to the predefined two-group criterion. Following this filtering step, 395 molecular features were retained for downstream statistical analyses.

Analytical preprocessing performed at this stage was distinguished from the data-dependent preprocessing subsequently used for machine-learning model development. Specifically, missing-value imputation and algorithm-specific feature standardization used for machine learning (ML) were performed within the corresponding cross-validation training folds, as described in Section 2.9, to prevent information from validation observations contributing to model preprocessing.

### 2.7. Statistical and Multivariate Analysis

Multivariate analyses were performed to explore global metabolic differences between the sPTB and term groups. Principal component analysis (PCA) was used as an unsupervised approach to examine the overall structure of the metabolomic data, identify potential outliers, and visualize clustering patterns. Partial least squares-discriminant analysis (PLS-DA) was subsequently applied as a supervised exploratory approach to assess group separation and estimate Variable Importance in Projection (VIP) scores [22]. PLS-DA was performed using the MetaboAnalyst 6.0 platform [23], and model performance was evaluated using 5-fold cross-validation, from which R^2^Y and Q^2^Y were obtained. Model significance was additionally assessed using a permutation test with 100 random permutations of the class labels. The permutation procedure compared the observed classification statistic with those obtained after random class-label permutation, yielding an empirical *p*-value of 0.01 (1/100 permutations).

Univariate comparisons of individual molecular features between the sPTB and term groups were performed using the Mann–Whitney U test. A nominal *p*-value <0.05 was used to identify candidate metabolites for subsequent exploratory analyses [24]. To account for multiple testing, *p*-values were additionally adjusted using the Benjamini–Hochberg false discovery rate (FDR) procedure [25]. Individual receiver operating characteristic (ROC) curves were constructed to characterize the discriminatory ability of selected metabolites, and the corresponding areas under the ROC curve (AUCs) were calculated [22,26].

For subsequent ML modeling, 30 metabolites showing nominal differences between groups (*p* < 0.05) were retained as a reduced metabolomic predictor set. The 30 metabolomic features were selected based on the preceding univariate analysis conducted in the complete analytical cohort prior to model development. Consequently, this initial metabolite selection was performed before the nested cross-validation procedure and was not repeated within each outer training fold.

### 2.8. Metabolite Annotation

Metabolite annotation was performed based on accurate mass, molecular formula, retention time, and MS/MS fragmentation patterns. Candidate metabolites were compared with the Human Metabolome Database (HMDB) [27] and Ceu Mass Mediator 3.0 [28]. Metabolites were classified according to the Metabolomics Standards Initiative (MSI) [29]. Annotation confidence was further supported using MS-DIAL version 5.5.250221 [30], LipidAnnotator [31], and Sirius 4 software platforms [32], through accurate-mass matching, spectral matching, lipid-specific annotation, and MS/MS-based molecular formula and structural prediction (Supplementary Table S1). Accordingly, level 2 annotations were supported by available MS/MS and spectral evidence, whereas level 3 annotations represent putative compound-class assignments with lower structural specificity. Because authentic reference standards were not analyzed, none of the reported metabolites should be considered definitively identified at MSI level 1 [29].

### 2.9. Machine Learning Modeling

#### 2.9.1. Feature Selection and Predictor Sets

Prior to ML modeling, a univariate feature-filtering step was performed using the Mann–Whitney U test, and metabolites showing nominal differences between the sPTB and term groups (*p* < 0.05) were retained for subsequent modeling [13,16]. This procedure resulted in the selection of 30 metabolomic features. The feature-selection step was performed using the complete analytical cohort before nested cross-validation and was not repeated within the outer-training folds [33].

Three predictor sets were evaluated for each ML algorithm: (i) clinical data, comprising maternal age, body mass index (BMI), history of previous preterm birth, cervical length, and cervical consistency index (CCI); (ii) metabolomic data, comprising the 30 selected metabolomic features; and (iii) integrated data, comprising the same 30 metabolomic features together with the five clinical variables. Gestational age at delivery was not included as a predictor because it was used to define the study outcome (sPTB versus term birth).

#### 2.9.2. Machine-Learning Algorithms and Preprocessing

Six supervised ML algorithms were evaluated: partial least squares-discriminant analysis (PLS-DA), logistic regression (LR), support vector machine (SVM), decision tree (DT), random forest (RF), and Extreme Gradient Boosting (XGBoost). PLS-DA was implemented using the PLSRegression class with binary-encoded class labels. PLS-DA, LR, SVM, DT, and RF were implemented in Python using the scikit-learn library [34], whereas XGBoost was implemented using the XGBoost Python package (version 2.1.2) [35]. The Python scripts used for model implementation and evaluation were developed and adapted following the methodological framework described by Han and collaborators [16].

Data-dependent preprocessing was incorporated within the modeling pipeline [25,34]. Missing metabolomic values were handled using K-nearest-neighbor (KNN) imputation (KNNImputer, k = 5). Within each cross-validation iteration, the imputer was fitted exclusively to the corresponding training data and subsequently applied unchanged to the validation data. Standardization using z-score transformation was performed within the training pipeline for algorithms sensitive to feature scale, including LR, SVM, and PLS-DA. Tree-based algorithms (DT, RF, and XGBoost) were not standardized.

Class imbalance was handled using algorithm-specific weighting when supported by the estimator [36]. Balanced class weights were used for LR, SVM, and DT; RF used balanced subsample weighting; and the XGBoost scale_pos_weight parameter was calculated separately within each outer-training set as the ratio of term to sPTB observations. No random oversampling, undersampling, or synthetic sample generation was performed. Class weighting was not applied to PLS-DA.

For SVM, hyperparameters were optimized within the inner cross-validation procedure, and the selected estimator was subsequently calibrated using sigmoid calibration restricted to the outer-training data [37]. For PLS-DA, two latent components were prespecified and were not optimized based on outcome performance. Missing-value imputation remained embedded within the corresponding cross-validation procedure [38,39]. The optimized hyperparameters for each algorithm are reported in Supplementary Table S4.

#### 2.9.3. Nested Cross-Validation and Model Development

Following the initial metabolite preselection described above, model development, hyperparameter optimization, and performance estimation were performed using nested stratified cross-validation [40,41]. A fivefold stratified outer cross-validation loop was used for model evaluation, while a separate fivefold stratified inner cross-validation loop was used exclusively for hyperparameter optimization. The same outer-fold partitions were used across all algorithms and predictor sets [41].

In each outer iteration, approximately 80% of participants constituted the outer-training set and the remaining 20% constituted the outer-validation fold. Hyperparameter optimization was performed exclusively within the corresponding outer-training data using fivefold stratified inner cross-validation, with ROC–AUC as the optimization criterion. The selected model was subsequently fitted using the outer-training data and evaluated on the corresponding held-out outer-validation fold. This procedure was repeated across the five outer folds, yielding one out-of-fold (OOF) prediction for each of the 199 participants [40].

#### 2.9.4. Model Performance and Statistical Comparison

Model performance was assessed using the area under the receiver operating characteristic curve (ROC–AUC), area under the precision–recall curve (PR–AUC), accuracy, balanced accuracy, sensitivity (recall), specificity, precision (positive predictive value), F1-score, and Matthews correlation coefficient (MCC) [42]. Primary performance estimates were summarized as the mean and standard deviation across the five outer validation folds. Predictions generated for the held-out participants in each outer fold were additionally concatenated to obtain one OOF prediction for each of the 199 participants. Aggregated OOF predictions were used to calculate complementary performance estimates and construct ROC curves and confusion matrices.

Uncertainty around the aggregated OOF performance estimates was quantified using 5000 participant-level stratified bootstrap resamples. Participants were resampled with replacement while preserving outcome stratification, and the corresponding performance metrics were recalculated. Percentile-based 95% confidence intervals (95% CIs) were obtained from the resulting bootstrap distributions [39].

Pairwise ROC–AUC comparisons were performed within each algorithm for three prespecified contrasts: integrated versus clinical, integrated versus metabolomic, and metabolomic versus clinical models. Comparisons were based on paired participant-level OOF predictions using 5000 paired stratified bootstrap resamples [43,44]. Within each bootstrap resample, the same participant indices were used for both models being compared. Differences in ROC–AUC (ΔAUC) and their percentile-based 95% CIs were calculated from the bootstrap distributions [33,44]. Two-sided bootstrap *p*-values were estimated from the proportion of resampled ΔAUC values on either side of zero. Because 18 pairwise comparisons were performed across the six algorithms, *p*-values were adjusted using the Benjamini–Hochberg false discovery rate (FDR) procedure. Bonferroni-adjusted *p*-values were additionally calculated as a sensitivity analysis [25].

Given the retrospective case–control design, precision and accuracy were interpreted as sample-specific classification metrics [45,46]. Model performance was therefore characterized using multiple complementary measures, including ROC–AUC, sensitivity, specificity, balanced accuracy, and MCC [42]. A classification threshold of 0.50 was used for binary classification metrics. For PLS-DA, ROC–AUC and PR–AUC were calculated from the continuous PLS response scores, whereas binary classification metrics were calculated using a cutoff of 0.50 applied to the continuous PLS response.

## 3. Results

### 3.1. Clinical and Obstetric Characteristics

A total of 199 pregnant women were included in the study, comprising 64 cases of spontaneous preterm birth (sPTB) and 135 term birth controls. Regarding the gestational-age distribution of the 64 sPTB cases, 5 (7.8%) deliveries occurred before 28 weeks, none between 28 and 31 + 6 weeks, 3 (4.7%) between 32 and 33 + 6 weeks, and 56 (87.5%) between 34 and 36 + 6 weeks. Thus, most sPTB cases in this cohort occurred during the late-preterm period. The clinical and obstetric characteristics of the study population are summarized in Table 1.

Most clinical and obstetric characteristics were comparable between the sPTB and term groups. Maternal age was similar between groups, with a median of 27.0 years (IQR: 24.3–31.0) in the sPTB group and 28.0 years (IQR: 24.0–30.0) in the term group (*p* = 0.8162). Likewise, no significant differences were observed in BMI (26.72 [IQR: 22.92–29.91] vs. 25.95 kg/m^2^ [IQR: 23.14–28.23]; *p* = 0.4818), cervical length (35 [IQR: 33–37] vs. 36 mm [IQR: 33–38]; *p* = 0.3308), or cervical consistency index (CCI) (0.809 [IQR: 0.769–0.874] vs. 0.833 [IQR: 0.784–0.871]; *p* = 0.4437) for the sPTB and term groups, respectively. Smoking history was also comparable between groups (6.3% vs. 9.6%; OR = 0.63, 95% CI: 0.22–1.99; *p* = 0.5892).

In contrast, significant differences were observed in gestational age at delivery and history of previous preterm birth. Women in the sPTB group had a significantly lower median gestational age at delivery than those in the term group (36.2 weeks [IQR: 34.5–36.5] vs. 39.0 weeks [IQR: 38.5–39.4]; *p* < 0.0001), as expected based on the study-group definitions. Moreover, a history of previous preterm birth was more frequent among women in the sPTB group than among the term controls (17.2% vs. 5.9%), and was significantly associated with sPTB in the current pregnancy (OR = 3.29, 95% CI: 1.25–8.65; *p* = 0.0116).

### 3.2. Multivariate Analysis of Metabolomic Data

To explore global metabolic variation between groups, principal component analysis (PCA) was performed as an unsupervised approach. The PCA score plot (Figure 1A) showed a partial separation between the sPTB and term groups, indicating slight differences in metabolomic profiles. However, a notable degree of overlap between groups was observed, reflecting the inherent biological variability and multifactorial nature of preterm birth.

Despite this overlap, the PCA distribution suggested that metabolic alterations are present early in pregnancy, although not sufficiently strong to achieve complete group discrimination using unsupervised methods alone. To further explore group discrimination, partial least squares-discriminant analysis (PLS-DA) was applied. As shown in Figure 1B, the supervised model showed a more apparent separation between the sPTB and term groups compared with the unsupervised PCA. However, the PLS-DA model yielded an R^2^Y value of 0.171 and a Q^2^Y value of 0.085, indicating that the model explained a limited proportion of the class-related variation and exhibited low predictive performance [38]. Thus, although supervised modeling revealed some structures associated with group membership, the PLS-DA results alone did not support robust discrimination between sPTB and term pregnancies [47].

These findings suggest that the metabolic differences associated with sPTB may not be adequately captured by conventional linear multivariate approaches such as PLS-DA. Given the high dimensionality and biological complexity of metabolomic data, group discrimination may involve more complex, potentially nonlinear relationships and interactions among metabolites and clinical variables. Therefore, machine-learning algorithms were subsequently evaluated to determine whether models capable of capturing more complex data structures could improve the discrimination between sPTB and term pregnancies.

### 3.3. Differential Metabolite Profiling

Thirty metabolites showed nominally significant differences between the sPTB and term groups (*p* < 0.05). After Benjamini–Hochberg FDR correction, six metabolites remained statistically significant. Given the exploratory nature of this study and the limited number of associations retained after multiple-testing correction, the remaining nominally significant metabolites were considered hypothesis-generating candidate signals and should not be interpreted as validated biomarkers. Lipid-related metabolites represented the predominant fraction of altered compounds. Specifically, lysophospholipids accounted for the largest proportion (28.6%), followed by phospholipids (17.9%). Other lipid-associated classes included lipid aldehydes (10.7%) and phosphatidylserines (10.7%), further supporting the prominence of lipid metabolism alterations. Additional contributions were observed from acylcarnitines (7.1%), sphingolipids (7.1%), and a group categorized as other metabolites (7.1%). Minor proportions corresponded to amino acid derivatives (3.6%), fatty acids and related compounds (3.6%), and glycerolipids (3.6%). Overall, the metabolic differentiation between the sPTB and term groups was driven primarily by lipid-associated metabolites, with non-lipid classes playing a minor role.

### 3.4. Discriminatory Capacity of Identified Metabolites

The predictive performance of the most discriminative annotated metabolites was evaluated using receiver operating characteristic (ROC) curve analysis. As shown in Figure 2, the top six metabolites exhibited moderate discriminatory capacity between the sPTB and term groups. The corresponding area under the curve (AUC) values indicated moderate to high classification performance, supporting their potential utility as candidate biomarkers. Among these, LPC O-14:0 exhibited the highest classification performance, with an AUC of 0.705, indicating the strongest individual discriminative ability within the evaluated metabolites. Similarly, PS 22:0 showed comparable discriminatory performance, with an AUC of 0.692.

ROC curves showed the individual predictive performance of the six annotated metabolites with the highest discriminatory capacity between sPTB and term birth. Other metabolites, including phosphorylcholine, tryptophanol, and MG 16:0, demonstrated slightly lower and relatively low discriminatory performance, with AUC values of 0.647, 0.653, and 0.643, respectively. In contrast, nonadec-enoylcarnitine showed the lowest discriminative capacity among the evaluated metabolites, with an AUC of 0.639 (Figure 2).

### 3.5. Exploratory Machine-Learning Model Performance

Machine-learning performance varied across algorithms and predictor sets (Table 2). Models based on clinical data showed limited discrimination, whereas models based on metabolomic and integrated data generally showed higher apparent ROC–AUC estimates. ROC curves generated from aggregated out-of-fold (OOF) predictions are presented separately for models based on clinical data (Figure 3), metabolomic data (Figure 4), and integrated clinical and metabolomic data (Figure 5). The corresponding OOF confusion matrices are presented in Supplementary Figures S1–S3.

#### 3.5.1. Models Based on Clinical Data

Models based exclusively on the five clinical variables showed limited discrimination between subsequent sPTB and term birth (Figure 3). Mean ROC–AUC values across the five outer validation folds were 0.505 ± 0.090 for XGBoost, 0.530 ± 0.075 for RF, 0.531 ± 0.044 for LR, 0.557 ± 0.083 for SVM, 0.514 ± 0.078 for DT, and 0.538 ± 0.045 for PLS-DA. Aggregated OOF estimates were similarly modest, with ROC–AUC values ranging from 0.500 to 0.555 across the evaluated algorithms. Specifically, OOF ROC–AUCs were 0.500 (95% CI: 0.412–0.587) for XGBoost, 0.529 (95% CI: 0.441–0.617) for RF, 0.555 (95% CI: 0.464–0.641) for LR, 0.511 (95% CI: 0.426–0.598) for SVM, 0.524 (95% CI: 0.440–0.609) for DT, and 0.546 (95% CI: 0.456–0.631) for PLS-DA (Table 2 and Figure 3).

Threshold-dependent metrics further reflected the limited ability of models based on clinical data to identify sPTB cases at the prespecified classification threshold of 0.50. XGBoost showed an OOF sensitivity of 0.469 and specificity of 0.548, correctly identifying 30 sPTB cases and 74 term births, with 34 false negatives and 61 false positives (Supplementary Figure S1). The corresponding balanced accuracy was 0.508 (Supplementary Table S2). RF showed lower sensitivity (0.281) but higher specificity (0.711), whereas LR yielded a sensitivity of 0.391 and specificity of 0.704 (Table 2). Among the ML algorithms, SVM showed the most pronounced imbalance between sensitivity and specificity. PLS-DA also showed a marked imbalance, with sensitivity and specificity values of 0.141 and 0.941, respectively, correctly identifying 9 sPTB cases and 127 term births (Supplementary Figure S1).

This threshold-dependent behavior was particularly evident for SVM. Although the continuous OOF scores yielded a ROC–AUC of 0.511, applying the prespecified classification threshold of 0.50 resulted in all 199 participants being classified as term births, yielding a sensitivity of 0 and a specificity of 1.00 (Supplementary Figure S1). This finding illustrates the distinction between discrimination based on continuous model scores and classification performance at a specific threshold, supporting the joint interpretation of ROC–AUC, sensitivity, specificity, balanced accuracy, and confusion matrices.

#### 3.5.2. Models Based on Metabolomic Data

In the exploratory analysis, models based on the 30 selected metabolomic features yielded higher ROC–AUC estimates than clinical-data models across most algorithms (Figure 4). Mean ROC–AUC values across the five outer validation folds were 0.767 ± 0.062 for RF, 0.762 ± 0.021 for XGBoost, 0.713 ± 0.018 for PLS-DA, 0.694 ± 0.057 for LR, 0.674 ± 0.115 for SVM, and 0.632 ± 0.070 for DT. Aggregated OOF estimates followed a similar pattern, with ROC–AUC values of 0.760 (95% CI: 0.687–0.827) for RF, 0.746 (95% CI: 0.667–0.817) for XGBoost, 0.716 (95% CI: 0.633–0.789) for PLS-DA, 0.705 (95% CI: 0.626–0.780) for SVM, 0.668 (95% CI: 0.581–0.750) for LR, and 0.632 (95% CI: 0.550–0.709) for DT (Table 2 and Figure 4).

Among the models based on metabolomic data, RF yielded an OOF sensitivity of 0.563 and specificity of 0.778, correctly identifying 36 sPTB cases and 105 term births, with 28 false negatives and 30 false positives (Supplementary Figure S2). Across the five outer validation folds, RF achieved a mean PR–AUC of 0.669 ± 0.106 and a balanced accuracy of 0.670 ± 0.052 (Supplementary Table S2). XGBoost showed an OOF sensitivity of 0.578, specificity of 0.785, precision of 0.561, F1-score of 0.569, and MCC of 0.360 (Table 2). Its mean PR–AUC and balanced accuracy across the outer validation folds were 0.646 ± 0.065 and 0.682 ± 0.050, respectively (Supplementary Table S2). The corresponding OOF confusion matrix included 37 true positives, 27 false negatives, 106 true negatives, and 29 false positives (Supplementary Figure S2).

At the prespecified threshold, LR showed a more balanced sensitivity–specificity profile, with an OOF sensitivity of 0.609 and specificity of 0.681. It correctly identified 39 sPTB cases and 92 term births, with 25 false negatives and 43 false positives (Table 2 and Supplementary Figure S2). In contrast, PLS-DA showed markedly higher specificity (0.933) than sensitivity (0.297), correctly identifying 19 sPTB cases and 126 term births (Supplementary Figure S2). Despite this threshold-dependent behavior, its continuous OOF scores yielded a ROC–AUC of 0.716, while the mean PR–AUC across the outer validation folds was 0.631 ± 0.063 (Supplementary Table S2).

A similar pattern was observed for SVM. Although its continuous OOF scores yielded a ROC–AUC of 0.705, sensitivity at the 0.50 threshold was 0.109, compared with a specificity of 0.956. Accordingly, 7 out of 64 sPTB cases and 129 out of 135 term births were correctly classified (Supplementary Figure S2). DT showed lower discrimination, with an OOF ROC–AUC of 0.632 and 28 true positives, 36 false negatives, 98 true negatives, and 37 false positives (Supplementary Figure S2).

#### 3.5.3. Models Integrating Clinical and Metabolomic Data

Models integrating the 30 selected metabolomic features with the five clinical variables yielded OOF ROC–AUC estimates ranging from 0.641 to 0.770 (Figure 5). Mean ROC–AUC values across the five outer validation folds were 0.777 ± 0.053 for RF, 0.768 ± 0.027 for LR, 0.755 ± 0.022 for XGBoost, 0.741 ± 0.034 for PLS-DA, 0.737 ± 0.045 for SVM, and 0.637 ± 0.057 for DT. Aggregated OOF estimates followed a similar pattern, with ROC–AUC values of 0.770 (95% CI: 0.699–0.836) for RF, 0.748 (95% CI: 0.672–0.818) for XGBoost, 0.743 (95% CI: 0.665–0.816) for LR, 0.741 (95% CI: 0.664–0.811) for PLS-DA, 0.738 (95% CI: 0.664–0.807) for SVM, and 0.641 (95% CI: 0.558–0.722) for DT (Table 2 and Figure 5).

Within the integrated models, RF showed a mean sensitivity of 0.528 ± 0.139 and specificity of 0.837 ± 0.056 across the five outer validation folds, with an MCC of 0.382 ± 0.105 (Table 2). Its mean PR–AUC was 0.670 ± 0.083, and balanced accuracy was 0.683 ± 0.060 (Supplementary Table S2). The aggregated OOF confusion matrix included 34 true positives, 30 false negatives, 113 true negatives, and 22 false positives (Supplementary Figure S3). XGBoost showed a mean sensitivity of 0.610 ± 0.071, specificity of 0.733 ± 0.071, precision of 0.525 ± 0.074, F1-score of 0.562 ± 0.062, and MCC of 0.333 ± 0.102 (Table 2). Its mean PR–AUC was 0.630 ± 0.068, with a balanced accuracy of 0.672 ± 0.051 (Supplementary Table S2). The aggregated OOF confusion matrix included 39 true positives, 25 false negatives, 99 true negatives, and 36 false positives (Supplementary Figure S3).

LR yielded an aggregated OOF ROC-AUC of 0.743. Across the five outer validation folds, mean sensitivity and specificity were 0.673 ± 0.135 and 0.770 ± 0.061, respectively (Table 2), while mean PR–AUC was 0.655 ± 0.058 and balanced accuracy was 0.722 ± 0.053 (Supplementary Table S2). At the prespecified threshold, the aggregated OOF predictions correctly classified 43 out of 64 sPTB cases and 104 out of 135 term births, with 21 false negatives and 31 false positives (Supplementary Figure S3).

PLS-DA yielded an aggregated OOF ROC–AUC of 0.741. Across the outer validation folds, mean sensitivity was 0.345 ± 0.052 and specificity was 0.919 ± 0.048 (Table 2), with a mean PR–AUC of 0.642 ± 0.079 (Supplementary Table S2). At the prespecified threshold of 0.50, the aggregated OOF predictions included 22 true positives, 42 false negatives, 124 true negatives, and 11 false positives (Supplementary Figure S3). Thus, the continuous PLS-DA scores showed greater ranking discrimination than might be inferred from sensitivity alone at the selected classification threshold.

A similar threshold-dependent pattern was observed for SVM. Although its aggregated OOF predictions yielded a ROC–AUC of 0.738, only 14 out of 64 sPTB cases were correctly classified at the 0.50 threshold, compared with 131 out of 135 term births, resulting in 50 false negatives and 4 false positives (Supplementary Figure S3). DT showed lower discrimination, with an OOF ROC–AUC of 0.641 and 34 true positives, 30 false negatives, 86 true negatives, and 49 false positives (Supplementary Figure S3).

### 3.6. Comparison of Predictor Sets

To explore differences in discrimination across predictor sets, paired ROC–AUC comparisons were performed using participant-level out-of-fold (OOF) predictions generated after the initial metabolite preselection and subsequent nested cross-validation procedure. For each algorithm, three prespecified comparisons were evaluated: clinical versus integrated, metabolomic versus integrated, and metabolomic versus clinical models. Given the 18 pairwise comparisons, *p*-values were adjusted using the Benjamini–Hochberg false discovery rate (FDR) procedure. Complete results, including unadjusted *p*-values and FDR-adjusted q-values, are provided in Supplementary Table S3.

Integrated models generally showed higher apparent ROC–AUC estimates than clinical models for most algorithms. The largest differences were observed for XGBoost (ΔAUC = 0.248, 95% CI: 0.128–0.362), RF (ΔAUC = 0.241, 95% CI: 0.128–0.353), SVM (ΔAUC = 0.227, 95% CI: 0.108–0.346), PLS-DA (ΔAUC = 0.195, 95% CI: 0.085–0.306), and LR (ΔAUC = 0.188, 95% CI: 0.076–0.298). These differences remained significant after FDR correction (q = 0.0009, 0.0009, 0.0009, 0.0041, and 0.0054, respectively). For DT, the difference was smaller (ΔAUC = 0.118, 95% CI: −0.001–0.235) and was not significant after FDR correction (q = 0.0786) (Supplementary Table S3).

Metabolomic models also showed higher ROC–AUC estimates than clinical models for XGBoost (ΔAUC = 0.246, 95% CI: 0.122–0.364; q = 0.0009), RF (ΔAUC = 0.231, 95% CI: 0.111–0.348; q = 0.0014), SVM (ΔAUC = 0.194, 95% CI: 0.083–0.301; q = 0.0036), and PLS-DA (ΔAUC = 0.170, 95% CI: 0.041–0.290; q = 0.0168). In contrast, the differences for LR (ΔAUC = 0.113, 95% CI: −0.021–0.244; q = 0.1250) and DT (ΔAUC = 0.108, 95% CI: −0.010–0.225; q = 0.0964) were not significant after FDR correction (Supplementary Table S3).

Adding the five clinical variables to the metabolomic predictors produced comparatively small changes in ROC–AUC (Figure 6). The differences between integrated and metabolomic models were 0.002 for XGBoost (95% CI: −0.054–0.059; q = 0.9324), 0.010 for RF (95% CI: −0.008–0.029; q = 0.3096), 0.075 for LR (95% CI: 0.006–0.150; q = 0.0605), 0.033 for SVM (95% CI: −0.035–0.103; q = 0.3654), 0.010 for DT (95% CI: −0.056–0.077; q = 0.8335), and 0.025 for PLS-DA (95% CI: approximately 0.000–0.052; q = 0.0786). None remained statistically significant after FDR correction.

LR showed the largest numerical increase in ROC–AUC after adding the clinical variables to the metabolomic predictor set (ΔAUC = 0.075, 95% CI: 0.006–0.150). Although this difference was nominally significant before adjustment for multiple comparisons (unadjusted *p* = 0.034), it did not remain significant after FDR correction (q = 0.0605) (Figure 6 and Supplementary Table S3). Accordingly, the increase observed for LR should be interpreted as a numerical difference rather than evidence of a statistically significant improvement in discrimination.

Overall, within this exploratory analysis, metabolomic models yielded higher ROC–AUC estimates than clinical models across several algorithms. However, adding the five clinical variables to the 30-metabolite predictor set did not significantly improve the ROC–AUC for any of the evaluated modeling approaches after correction for multiple comparisons. These comparisons should be interpreted cautiously because the metabolomic predictor set was selected using outcome-associated analysis in the complete cohort before cross-validation, and the resulting discrimination estimates may therefore be subject to some degree of overestimation.

## 4. Discussion

The present study identified first-trimester plasma metabolomic alterations associated with subsequent spontaneous preterm birth (sPTB) and explored their discriminatory capacity using multiple ML approaches within a nested cross-validation framework. Overall, three main findings emerged from this study. First, models based on maternal age, body mass index (BMI), history of previous preterm birth, cervical length, and cervical consistency index (CCI) showed limited discrimination in this cohort. Second, metabolomic profiles differed between pregnancies subsequently resulting in sPTB and those resulting in term birth, with lipid-related metabolites accounting for a substantial proportion of the altered molecular features. Third, metabolomic-data models generally showed moderate discrimination in the exploratory analysis, while the addition of the five clinical variables did not result in a statistically significant improvement in ROC–AUC after correction for multiple comparisons. Collectively, these findings suggest that first-trimester metabolomic profiles contain biochemical information relevant to the discrimination of subsequent sPTB beyond that captured by the limited set of clinical variables evaluated in this study.

Most of the clinical and obstetric characteristics evaluated in this cohort did not differ significantly between the sPTB and term groups. Maternal age, BMI, cervical length, and CCI showed comparable distributions, whereas a history of previous preterm birth was significantly associated with subsequent sPTB (OR = 3.29, 95% CI: 1.25–8.65). This finding is consistent with previous evidence identifying the history of spontaneous preterm birth as an important risk factor for recurrence [48,49]. In the present cohort, however, the limited discriminatory performance of models based on the five evaluated clinical variables suggests that these characteristics alone did not adequately distinguish pregnancies subsequently resulting in sPTB from those resulting in term birth. The metabolomic differences observed in the first trimester may therefore provide complementary information on biological alterations associated with subsequent sPTB.

This interpretation was further supported by the ML analyses. Across the six evaluated algorithms, models based on clinical data showed limited discrimination, with aggregated out-of-fold (OOF) ROC–AUC values ranging from approximately 0.50 to 0.56. Similarly, limited discrimination based on first-trimester clinical characteristics alone has been reported in pregnancies subsequently resulting in preterm and term delivery, whereas improved discrimination was observed when molecular information derived from Raman spectroscopy was incorporated [50]. However, differences in study design, predictor definitions, sample size, analytical platform, and validation strategy limit direct numerical comparisons with the present study.

A major finding was the predominance of lipid-related metabolites among the nominally associated molecular features. Lysophospholipids, phospholipids, lipid aldehydes, and phosphatidylserines represented a substantial proportion of these candidate metabolites, suggesting that alterations in lipid metabolism warrant further investigation in relation to sPTB. This pattern is consistent with previous studies linking lipid dysregulation to adverse pregnancy outcomes, including sPTB [2,51,52,53,54,55,56]. Lipid metabolism plays a central role in membrane structure, energy homeostasis, and signaling pathways, all of which are critical during pregnancy [57,58]. Importantly, lysophospholipids such as lysophosphatidylcholine (LPC) and lysophosphatidylethanolamine (LPE) are key intermediates in membrane remodeling and are generated through phospholipase A_2_ (PLA_2_) activity as part of the Lands cycle [59,60,61,62]. Their increased abundance in sPTB cases may be consistent with altered membrane turnover and inflammatory signaling, although these processes were not directly assessed in the current analysis.

Biologically, LPC species act as pro-inflammatory mediators capable of inducing cytokine and chemokine production, endothelial activation, and immune cell recruitment [60,63]. The consistent increase of LPC species observed in this study supports the hypothesis of early inflammatory activation in pregnancies that later develop sPTB.

Similarly, LPE species showed differential regulation, which has also been associated with inflammatory responses and cellular stress mechanisms [64,65]. Previous studies have reported similar patterns, including increased lysophospholipids in maternal plasma during pregnancy complicated by sPTB [2,66]. Particularly relevant to the present findings, the lysolipid 1-palmitoleoyl-GPE (16:1) was identified as the strongest individual metabolite predictor across several gestational time points, including the first trimester [13]. Although the specific molecular species identified across studies are not identical, the repeated observation of lysophospholipid alterations strengthens the rationale for the further investigation of membrane lipid remodeling in the early pathophysiology of sPTB. Beyond lysophospholipids, alterations in phosphatidylcholines (PCs) further support the involvement of membrane remodeling processes. PCs are major structural components of cellular membranes and act as key intermediates in lipid metabolism and signaling [58,64]. Their hydrolysis generates LPC and fatty acids, which can be further converted into eicosanoids, mediators known to regulate inflammation and uterine contractility [64]. In maternal–fetal tissues, lipid peroxidation has been associated with extracellular matrix degradation, activation of matrix metalloproteinases (MMPs), and increased prostaglandin production, all of which can contribute to premature labor onset [67]. Taken together, these findings indicate that sPTB may involve coordinated alterations in lipid metabolism, membrane remodeling, oxidative stress, and inflammatory signaling pathways. However, because the present study was observational and metabolite identities remain putative for several features, these mechanistic interpretations should be regarded as biologically plausible hypotheses rather than evidence of causal pathways.

Although the PLS-DA permutation test yielded a statistically significant result (*p* = 0.01), the model showed limited overall explanatory and cross-validated predictive performance. The R^2^Y value of 0.171 indicated that only a modest proportion of outcome-related variation was explained by the model, while the Q^2^Y value of 0.085 indicated low cross-validated predictive capacity. Accordingly, the apparent separation observed in the supervised score space should be interpreted cautiously and should not be considered evidence of strong classification performance. This finding also illustrates the importance of evaluating supervised metabolomic models using quantitative measures of generalization rather than relying on visual separation alone [2,68]. High-dimensional metabolomic datasets are particularly susceptible to overfitting when the number of candidate molecular features is large relative to the number of participants [69]. In contrast, studies with smaller and less variable cohorts have reported higher predictive values [70], suggesting that model performance is strongly influenced by sample heterogeneity.

The explorative ML analysis provided an assessment of the discriminatory information contained in the different predictor sets. Models based on metabolomic data generally showed moderate discrimination, with RF and XGBoost showing the highest ROC–AUC estimates among the evaluated approaches (Table 2). Aggregated OOF estimates showed a similar pattern (Figure 4). However, because metabolite selection was performed using the complete cohort before cross-validation, these estimates may be optimistic and should not be interpreted as fully validated estimates of predictive performance [71].

These findings are broadly consistent with the moderate performance reported in previous metabolomic research on sPTB. Among the 399 participants, including 98 sPTB cases, untargeted serum metabolomics combined with six supervised machine-learning approaches identified 47 metabolites considered relevant across multiple methods. A reduced four-predictor model yielded an optimism-corrected ROC–AUC of 0.703 at 28 weeks, whereas its application to samples collected at 12 weeks resulted in an AUC of 0.606 (95% CI: 0.544–0.667) [13]. Although differences in population characteristics, sampling time, analytical methods, and validation strategies preclude direct numerical comparisons, these findings support the presence of measurable metabolomic signals associated with subsequent sPTB while highlighting the challenges of achieving strong discrimination early in pregnancy.

The present study extends this evidence by evaluating first-trimester plasma from a Colombian cohort using untargeted LC-QTOF-MS and by comparing clinical, metabolomic, and integrated predictor sets within the same analytical framework. Metabolomic studies addressing early discrimination of sPTB remain limited in Latin American populations, and data from this Colombian cohort contribute to expanding the geographical representation of this research. Importantly, the present findings should be considered exploratory, and independent prospective validation with feature selection performed within the training folds will be required to assess their reproducibility and generalizability.

The relative contribution of metabolomic and clinical information observed in the present study should be interpreted in the context of previous molecular studies of preterm birth. Metabolomic investigations have reported associations between maternal molecular profiles and subsequent sPTB, although their discriminatory performance varied according to gestational age, biospecimen, analytical platform, feature-selection strategy, and validation approach [13,16,50,72]. In a case-cohort study of 399 pregnancies, including 98 sPTB cases, untargeted serum metabolomics combined with multiple supervised modeling approaches identified a predominantly lipid-related molecular signature, although discrimination was modest when the resulting model was applied to first-trimester samples [13]. Similarly, first-trimester plasma profiling using Raman spectroscopy and metabolomics demonstrated that molecular information contributed discriminatory information beyond that obtained from the clinical characteristics evaluated in that cohort [50]. Maternal serum and vaginal metabolomic profiles have also been associated with subsequent preterm birth, although the findings differed according to sampling time, biological matrix, predictor selection, and validation procedure [16,72]. More recently, comparative evaluation of multiple modeling approaches applied to maternal serum metabolomics showed that performance varied across algorithms and resampling strategies, highlighting the influence of the analytical framework on metabolomics-based classification [16].

Taken together, these findings indicate that the contributions of metabolomic and clinical information may vary across populations and analytical strategies. In the present study, the lack of statistically significant improvement in ROC–AUC after adding the five evaluated clinical variables should therefore be interpreted specifically within the current predictor set and modeling framework, rather than as evidence that clinical information has no value for sPTB discrimination.

Rather, these findings indicate that within the present dataset and modeling framework, the selected clinical variables did not provide substantial additional discriminatory information once the metabolomic predictors were included. A similar pattern has been reported in a nested cross-validation study of sPTB, in which a metabolomics-based model showed greater discrimination than a model based on clinical variables, while integration of clinical, microbiome, and metabolomic data did not significantly improve discrimination compared with metabolomics alone [73]. In contrast, other first-trimester studies have reported improved classification when molecular information was combined with maternal clinical and obstetric characteristics [50], suggesting that the incremental contribution of clinical information may vary across populations and analytical approaches. Moreover, multi-omics studies of preterm birth have shown that integration can improve discrimination when the combined molecular layers provide complementary information [74]. Collectively, these findings suggest that improved discrimination through data integration may depend not simply on adding more predictors, but on whether the additional variables provide information that is complementary to the molecular profile. Differences in the clinical variables selected, biological matrix, molecular platform, gestational age at sampling, population characteristics, and validation strategy may therefore influence the incremental value obtained from integrated models. Gestational timing may be particularly relevant because maternal metabolic profiles change dynamically throughout pregnancy [75].

## 5. Limitations

Several limitations should be acknowledged. This retrospective case–control design and modest sample size, including 64 sPTB cases, limit generalizability, and the study case fraction does not represent the prevalence of sPTB in the target population. Moreover, the sPTB group was predominantly composed of late-preterm births, with 56 out of 64 cases (87.5%) occurring between 34 + 0 and 36 + 6 weeks of gestation and only eight cases occurring before 34 weeks. Consequently, the metabolomic alterations and exploratory discriminatory estimates observed in this study predominantly reflect late sPTB and may not be generalizable to earlier preterm birth phenotypes. Future studies with larger and more balanced representation across gestational-age subgroups are needed to determine whether the metabolic alterations identified here are also observed in earlier forms of sPTB. Consequently, prevalence-dependent measures such as precision and PR–AUC should be interpreted as sample-specific rather than population-level estimates. External validation in prospective populations will therefore be necessary to assess calibration and clinical utility [76,77]. In addition, several metabolite annotations remain putative and require confirmation using authentic analytical standards.

Potential residual confounding should also be considered when interpreting the observed metabolomic associations [69]. Previous preterm birth was more frequent among women who subsequently experienced sPTB and may therefore have contributed to some of the observed metabolic differences. Although the study collected several maternal and obstetric characteristics, including gestational age at sampling, obstetric history, smoking history, recent antibiotic exposure, and previously diagnosed medical conditions, the present exploratory metabolomic associations were not comprehensively adjusted for these factors. In addition, fasting status, time of day of sample collection, and other potentially relevant preanalytical or clinical factors were not systematically characterized for the present analysis. Samples were collected during the first trimester (before 15 weeks of gestation), stored at −80 °C, and analyzed within the same analytical experiment; nevertheless, residual clinical and preanalytical confounding cannot be excluded. Future prospective studies should incorporate comprehensive covariate adjustment and standardized preanalytical assessment to determine whether the identified metabolomic associations are independent of these factors.

A major methodological limitation concerns the high dimensionality of the metabolomic dataset and the preselection of candidate metabolites. Although imputation, scaling, hyperparameter optimization, and model fitting were performed within the nested cross-validation framework, the 30 candidate metabolites were selected using outcome-associated univariate analyses performed on the complete analytical cohort before nested cross-validation. Consequently, outcome information from participants subsequently included in the outer-validation folds contributed to predictor selection, resulting in information leakage [78,79]. Therefore, the outer-validation folds do not represent fully independent evaluations of the complete feature-selection and modeling pipeline, and the reported performance estimates may be subject to some degree of overestimation. This limitation is particularly relevant given the high dimensionality of untargeted metabolomic data relative to the available sample size, which further increases susceptibility to overfitting [69,80]. Accordingly, the ML analyses should be interpreted as exploratory and hypothesis-generating rather than as evidence of validated predictive performance or clinical utility. Future studies should perform feature selection entirely within the training folds of the validation framework, evaluate feature-selection stability across folds, and subsequently validate the resulting models in independent prospective cohorts.

## 6. Conclusions

The identification of early metabolomic signatures may contribute to a better understanding of the biological alterations associated with subsequent sPTB. This study identified first-trimester plasma metabolomic differences associated with subsequent sPTB, predominantly involving lipid metabolism. Individual annotated metabolites showed moderate discriminatory capacity, while machine-learning models based on metabolomic data also showed moderate discrimination and generally higher ROC–AUC estimates than models based on the five evaluated clinical variables. However, adding these clinical variables to the metabolomic predictor set did not provide a statistically significant incremental improvement in ROC–AUC after correction for multiple comparisons. These findings support the further investigation of first-trimester metabolomic signatures as candidate markers associated with subsequent sPTB.

Nevertheless, these findings should be considered exploratory and hypothesis-generating. The retrospective case–control design, modest sample size, and absence of external validation limit the generalizability of the identified metabolic signatures and model-performance estimates. In addition, the 30 metabolomic predictors used for machine-learning modeling were preselected using outcome-associated univariate results from the complete analytical cohort before nested cross-validation, which may have introduced optimism into the estimated model performance. The observational design also precludes causal inference, and the structural identities of candidate metabolites require further confirmation using authentic analytical standards. Future studies should validate these metabolic signatures in larger prospective cohorts, incorporate feature selection entirely within the resampling framework, confirm metabolite identities, and evaluate model calibration and clinical utility before their potential application to sPTB risk assessment.

## Figures and Tables

**Figure 1:**
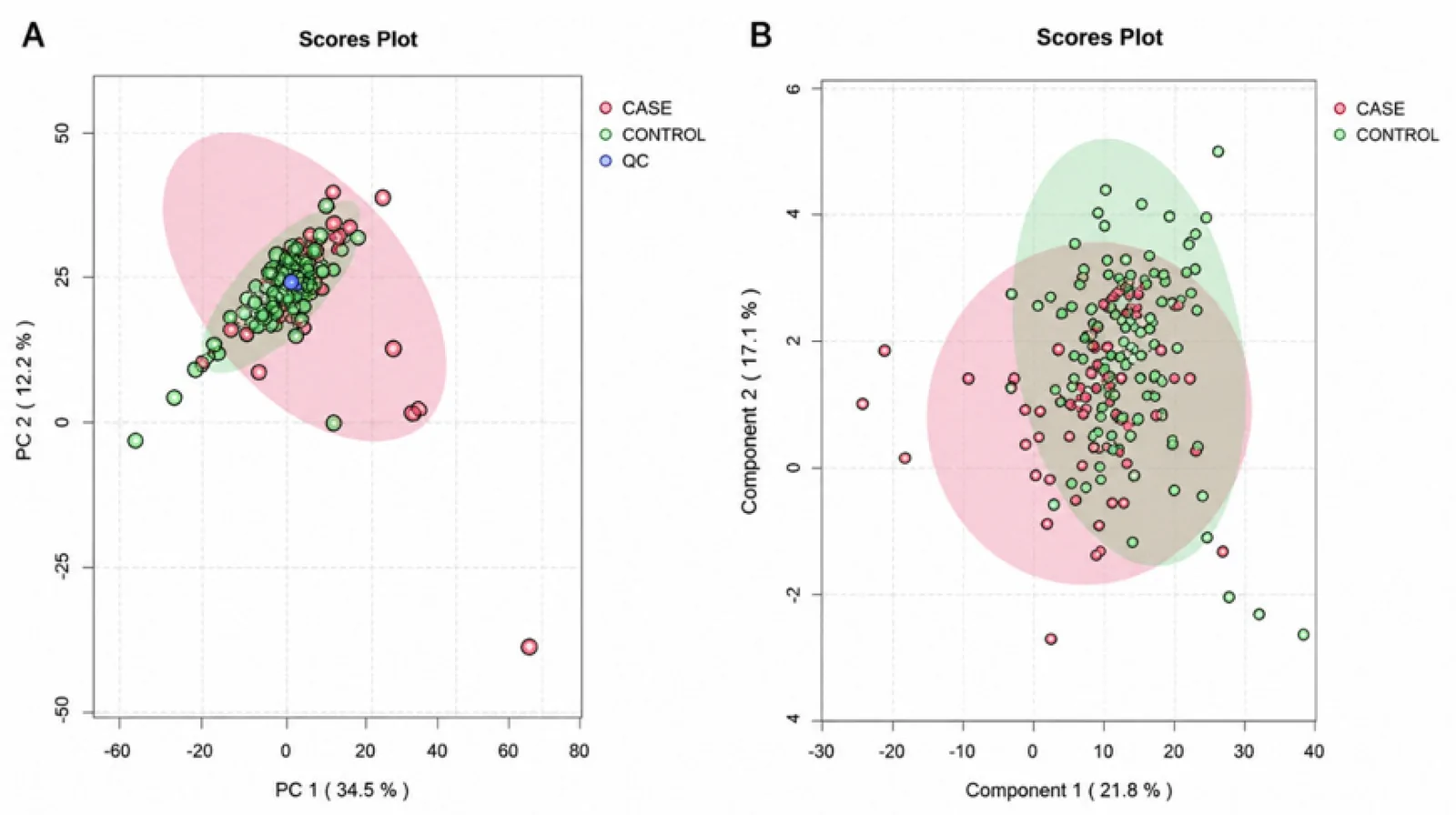
Multivariate analysis of plasma metabolomic profiles in women with spontaneous preterm birth (sPTB) and term birth. (**A**) Principal component analysis (PCA) score plot showing the distribution of plasma metabolomic profiles from women with sPTB and term birth. (**B**) Partial least squares-discriminant analysis (PLS-DA) score plot illustrating supervised discrimination between study groups. The PLS-DA model yielded an R^2^Y = 0.171 and Q^2^Y = 0.085, indicating limited predictive performance despite improved group separation compared with PCA.

**Figure 2:**
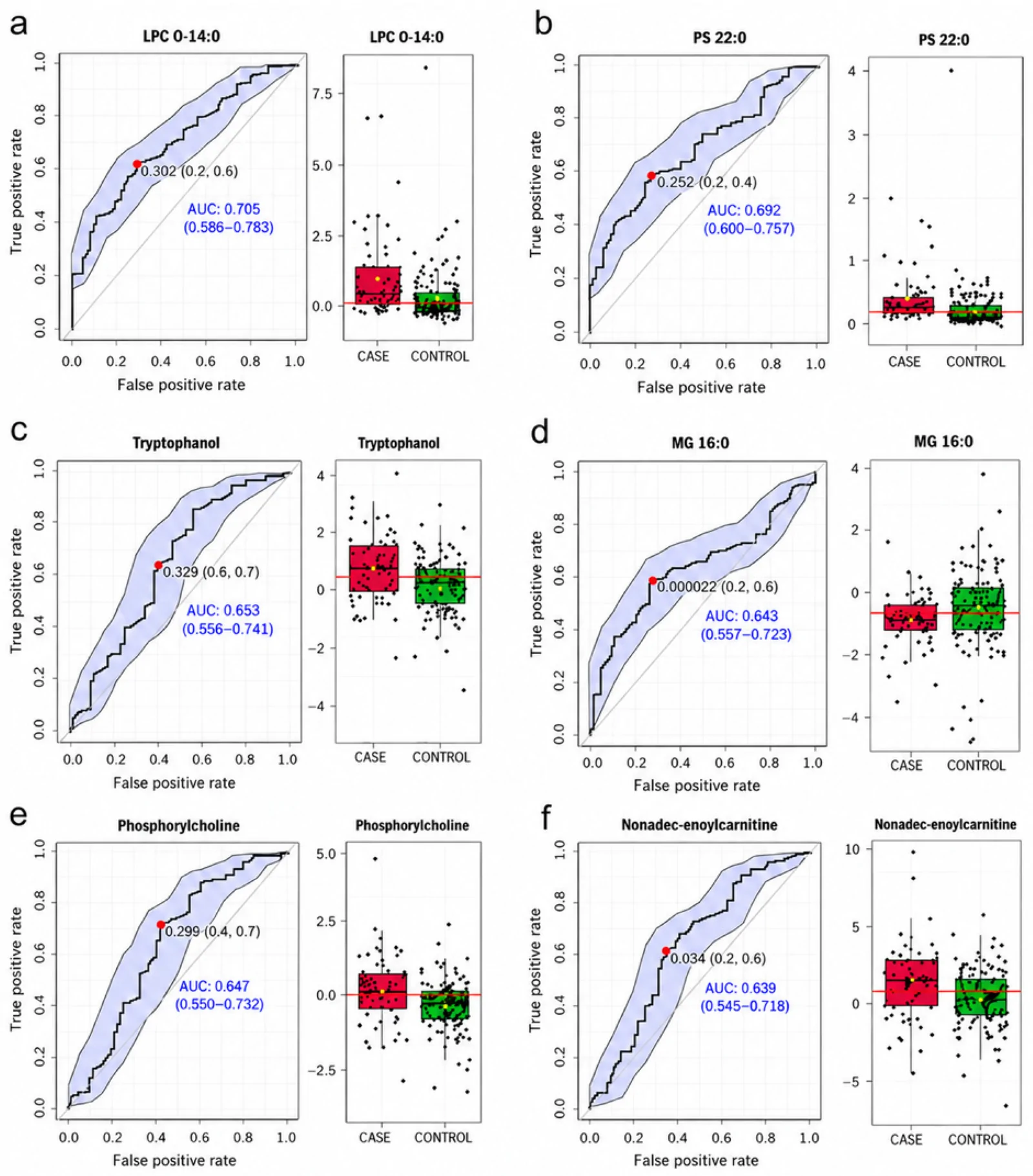
Receiver operating characteristic (ROC) curve analysis of the most discriminative annotated metabolites associated with preterm birth: (**a**) LPC O-14:0; (**b**) PS 22:0; (**c**) tryptophanol; (**d**) MG 16:0; (**e**) phosphorylcholine; and (**f**) nonadec-enoylcarnitine. In the box plots, red represents the case group and green represents the control group.

**Figure 3:**
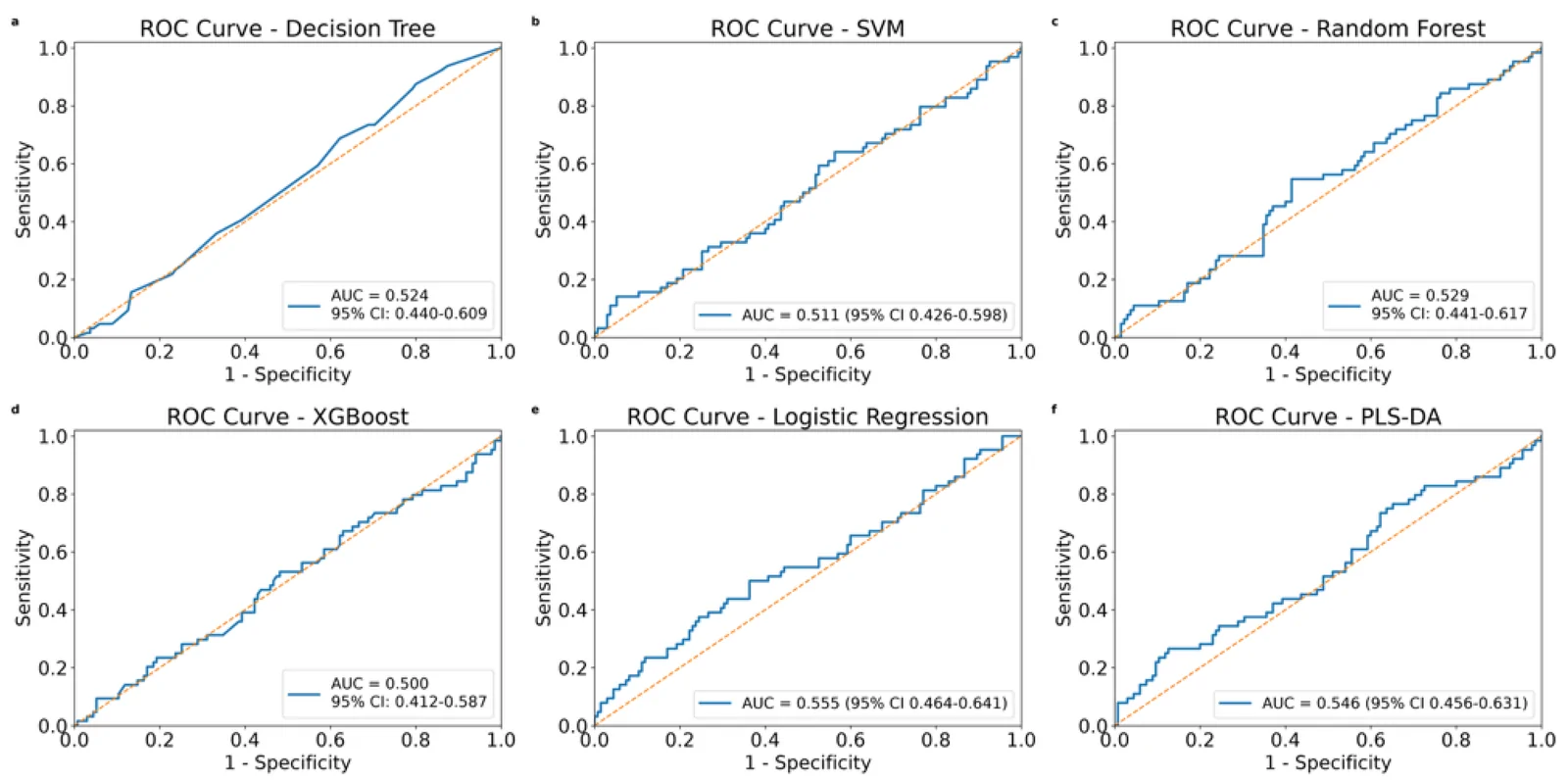
ROC curves for machine-learning models based on clinical data under nested cross-validation: (**a**) decision tree (DT); (**b**) support vector machine (SVM); (**c**) random forest (RF); (**d**) Extreme Gradient Boosting (XGBoost); (**e**) logistic regression (LR); and (**f**) partial least squares-discriminant analysis (PLS-DA). Receiver operating characteristic (ROC) curves were generated from aggregated out-of-fold (OOF) predictions obtained across the five outer validation folds. The clinical predictor set comprised maternal age, body mass index, history of previous preterm birth, cervical length, and cervical consistency index (CCI). Curves are shown for decision tree (DT), support vector machine (SVM), random forest (RF), Extreme Gradient Boosting (XGBoost), logistic regression (LR), and partial least squares-discriminant analysis (PLS-DA). AUC values and participant-level bootstrap 95% confidence intervals based on 5000 resamples are reported for each model.

**Figure 4:**
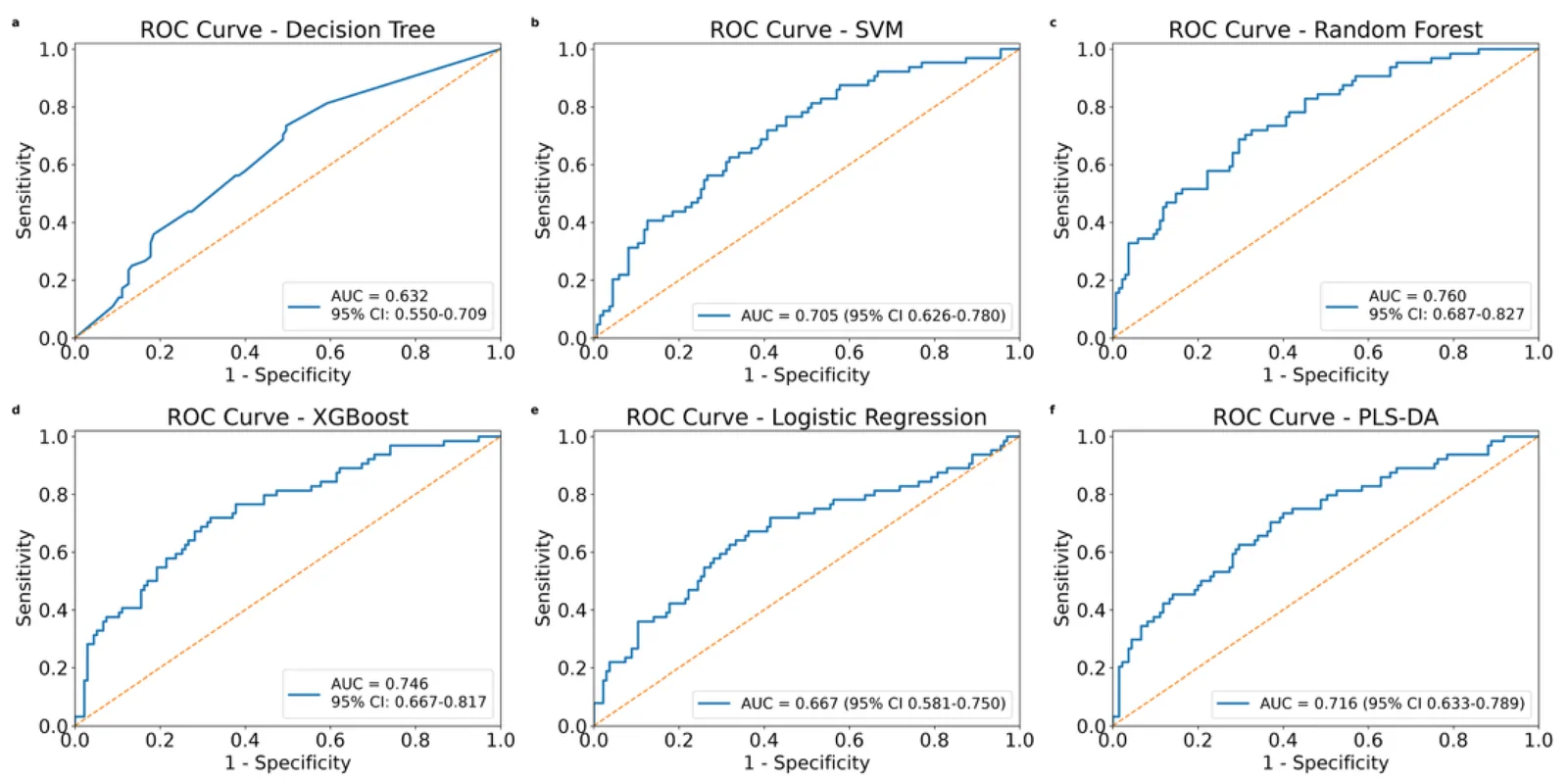
ROC curves for machine-learning models based on metabolomic data under nested cross-validation: (**a**) decision tree (DT); (**b**) support vector machine (SVM); (**c**) random forest (RF); (**d**) Extreme Gradient Boosting (XGBoost); (**e**) logistic regression (LR); and (**f**) partial least squares-discriminant analysis (PLS-DA). Receiver operating characteristic (ROC) curves were generated from aggregated out-of-fold (OOF) predictions obtained across the five outer validation folds. The metabolomic predictor set comprised the 30 preselected metabolomic features. Curves are shown for decision tree (DT), support vector machine (SVM), random forest (RF), Extreme Gradient Boosting (XGBoost), logistic regression (LR), and partial least squares-discriminant analysis (PLS-DA). AUC values and participant-level bootstrap 95% confidence intervals based on 5000 resamples are reported for each model.

**Figure 5:**
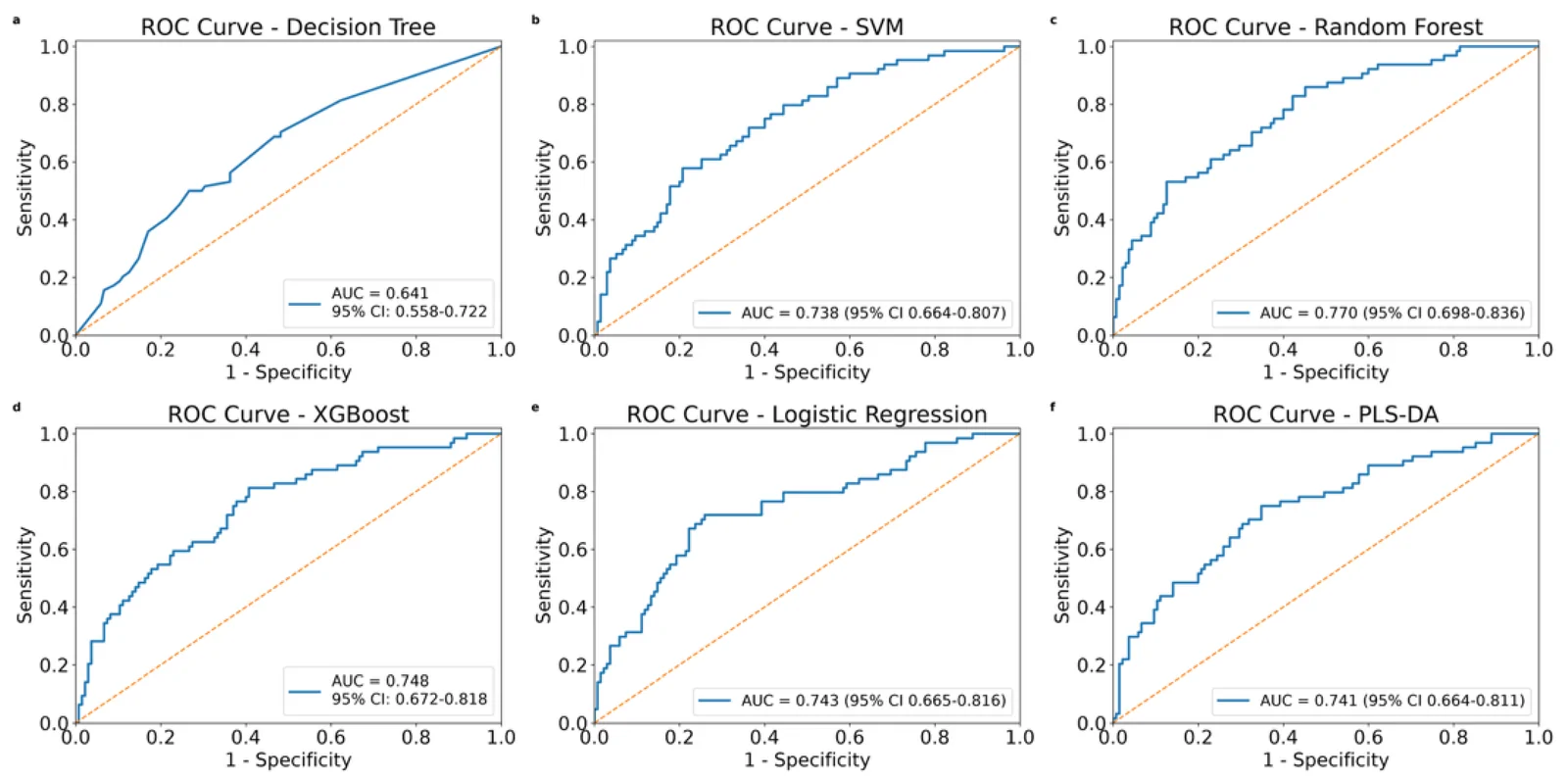
ROC curves for combined clinical-metabolomic machine-learning models under nested cross-validation: (**a**) decision tree (DT); (**b**) support vector machine (SVM); (**c**) random forest (RF); (**d**) Extreme Gradient Boosting (XGBoost); (**e**) logistic regression (LR); and (**f**) partial least squares-discriminant analysis (PLS-DA). Receiver operating characteristic (ROC) curves were generated from aggregated out-of-fold (OOF) predictions obtained across the five outer validation folds. The combined predictor set comprised the 30 preselected metabolomic features together with maternal age, body mass index, history of previous preterm birth, cervical length, and cervical consistency index (CCI). Curves are shown for decision tree (DT), support vector machine (SVM), random forest (RF), Extreme Gradient Boosting (XGBoost), logistic regression (LR), and partial least squares-discriminant analysis (PLS-DA). AUC values and participant-level bootstrap 95% confidence intervals (5000 iterations) are reported for each model.

**Figure 6:**
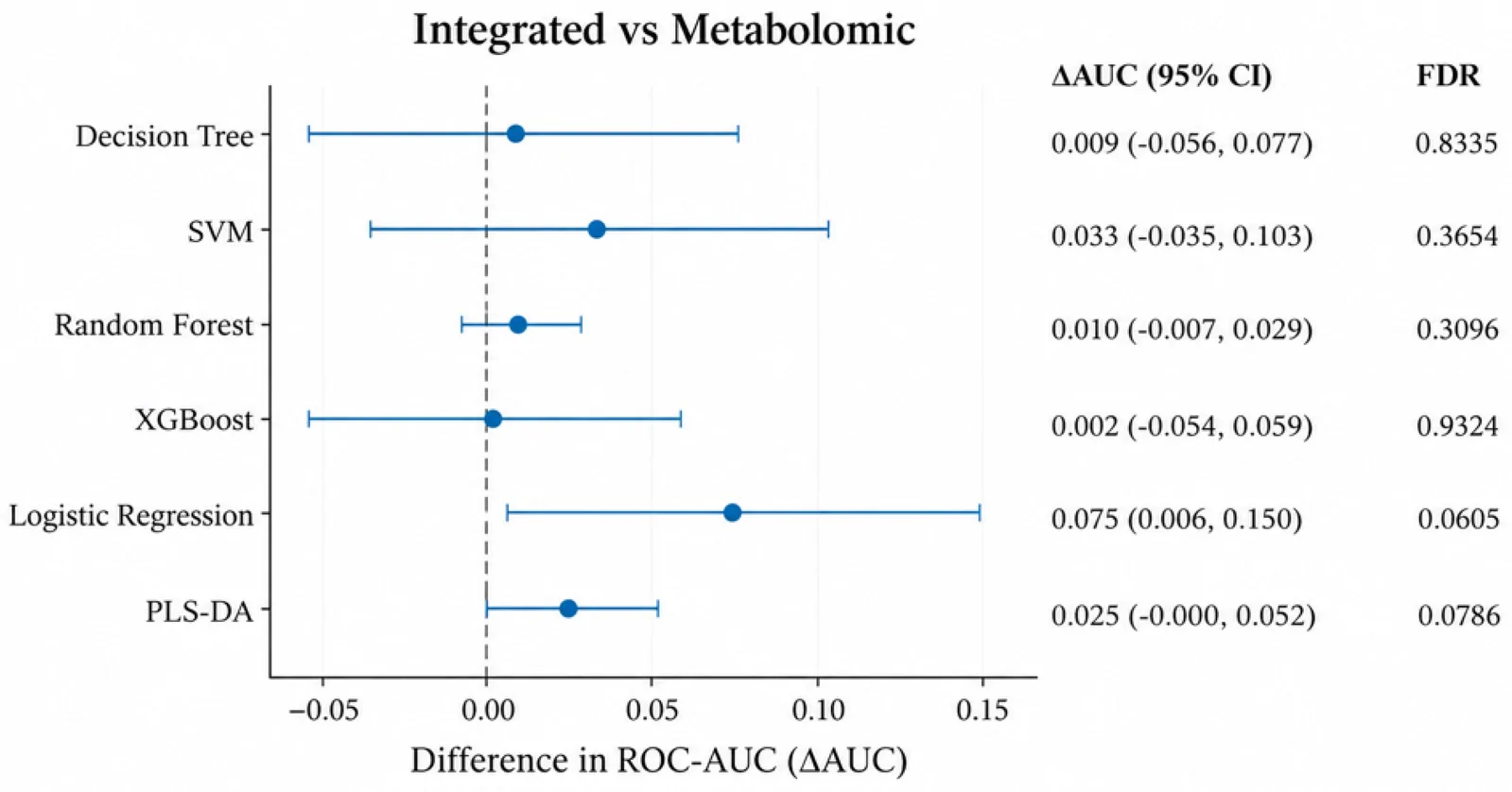
Paired differences in ROC–AUC between integrated and metabolomic machine-learning models. Differences in ROC–AUC (ΔAUC) were calculated from paired participant-level out-of-fold (OOF) predictions generated within the nested stratified cross-validation framework, with ΔAUC defined as AUC_integrated − AUC_metabolomic. Positive values indicate higher ROC–AUC estimates for the integrated configuration. Error bars represent percentile-based 95% confidence intervals obtained from 5000 paired stratified bootstrap resamples. The vertical dashed line indicates no difference in ROC–AUC (ΔAUC = 0). FDR-adjusted q-values were calculated using the Benjamini–Hochberg procedure across the 18 prespecified pairwise model comparisons.

**Table 1:** Clinical and obstetric characteristics of women with spontaneous preterm birth (sPTB) and term birth.

Characteristics	Women with Preterm Birth (N = 64) *	Women with Term Birth (N = 135) *	*p*-Value
Maternal age (years)	27.0 (24.3–31.0)	28.0 (24.0–30.0)	0.8162
$\mathrm{Body}\;\mathrm{mass}\;\mathrm{index}\;(\mathrm{BMI})\;(\mathrm{kg}/m^{2})$	26.72 (22.92–29.91)	25.95 (23.14–28.23)	0.4818
Gestational age at delivery (weeks)	36.2 (34.5–36.5)	39.0 (38.5–39.4)	<0.0001
Cervical length (mm)	35 (33–37)	36 (33–38)	0.3308
Cervical consistency index (CCI)	0.809 (0.769–0.874)	0.833 (0.784–0.871)	0.4437
Smoking history	OR = 0.63 (95% CI: 0.22–1.99)		0.5892
Smoker	4 (6.3%)	13 (9.6%)	
Non smoker	60 (93.7%)	122 (90.4%)	
History of preterm birth	OR = 3.29 (95% CI: 1.25–8.65)		0.0116
Yes	11 (17.2%)	8 (5.9%)	
No	53 (82.8%)	127 (94.1%)	

* Continuous variables are presented as median (interquartile range, IQR) and categorical variables as frequency (percentage). Comparisons between groups were performed using the Mann–Whitney U test for continuous variables and the chi-square test or Fisher’s exact test for categorical variables, as appropriate. Odds ratios (OR) and 95% confidence intervals (95% CI) are reported for categorical variables. Statistical significance was defined as *p* < 0.05. BMI, body mass index; CCI, cervical consistency index; sPTB, spontaneous preterm birth.

**Table 2:** Performance of machine-learning models across predictor sets using nested stratified cross-validation.

Algorithm	Predictor Data	ROC-AUC	Accuracy	Sensitivity (Recall)	Specificity	Precision (PPV)	F1 Score	MCC
XGBoost	Clinical	0.505 ± 0.090	0.523 ± 0.042	0.471 ± 0.186	0.548 ± 0.112	0.318 ± 0.064	0.373 ± 0.115	0.013 ± 0.110
	Metabolomic	0.762 ± 0.021	0.719 ± 0.031	0.578 ± 0.128	0.785 ± 0.048	0.560 ± 0.038	0.564 ± 0.077	0.361 ± 0.086
	Integrated	0.755 ± 0.022	0.693 ± 0.054	0.610 ± 0.071	0.733 ± 0.071	0.525 ± 0.074	0.562 ± 0.062	0.333 ± 0.102
Random Forest	Clinical	0.530 ± 0.075	0.573 ± 0.066	0.282 ± 0.091	0.711 ± 0.127	0.324 ± 0.047	0.293 ± 0.059	−0.005 ± 0.077
	Metabolomic	0.767 ± 0.062	0.708 ± 0.040	0.562 ± 0.093	0.778 ± 0.037	0.544 ± 0.065	0.551 ± 0.073	0.337 ± 0.099
	Integrated	0.777 ± 0.053	0.739 ± 0.042	0.528 ± 0.139	0.837 ± 0.056	0.612 ± 0.069	0.558 ± 0.097	0.382 ± 0.105
Logistic Regression	Clinical	0.531 ± 0.044	0.603 ± 0.087	0.390 ± 0.046	0.704 ± 0.134	0.407 ± 0.096	0.391 ± 0.046	0.102 ± 0.128
	Metabolomic	0.694 ± 0.057	0.659 ± 0.046	0.610 ± 0.178	0.681 ± 0.077	0.470 ± 0.066	0.526 ± 0.109	0.277 ± 0.131
	Integrated	0.768 ± 0.027	0.739 ± 0.036	0.673 ± 0.135	0.770 ± 0.061	0.584 ± 0.042	0.619 ± 0.071	0.431 ± 0.089
SVM	Clinical	0.557 ± 0.083	0.678 ± 0.008	0.000 ± 0.000	1.000 ± 0.000	0.000 ± 0.000	0.000 ± 0.000	0.000 ± 0.000
	Metabolomic	0.674 ± 0.115	0.683 ± 0.016	0.109 ± 0.116	0.956 ± 0.048	0.481 ± 0.364	0.162 ± 0.146	0.115 ± 0.116
	Integrated	0.737 ± 0.045	0.729 ± 0.032	0.218 ± 0.098	0.970 ± 0.048	0.864 ± 0.196	0.330 ± 0.125	0.321 ± 0.101
Decision Tree	Clinical	0.514 ± 0.078	0.543 ± 0.081	0.408 ± 0.255	0.607 ± 0.194	0.314 ± 0.073	0.335 ± 0.153	0.011 ± 0.147
	Metabolomic	0.632 ± 0.070	0.633 ± 0.052	0.440 ± 0.159	0.726 ± 0.113	0.438 ± 0.097	0.427 ± 0.096	0.169 ± 0.115
	Integrated	0.637 ± 0.057	0.602 ± 0.108	0.536 ± 0.175	0.637 ± 0.230	0.445 ± 0.108	0.463 ± 0.041	0.181 ± 0.106
PLS-DA	Clinical	0.538 ± 0.045	0.684 ± 0.026	0.140 ± 0.063	0.941 ± 0.042	0.600 ± 0.253	0.216 ± 0.077	0.148 ± 0.092
	Metabolomic	0.713 ± 0.018	0.729 ± 0.032	0.297 ± 0.039	0.933 ± 0.048	0.703 ± 0.126	0.413 ± 0.044	0.318 ± 0.081
	Integrated	0.741 ± 0.034	0.734 ± 0.033	0.345 ± 0.052	0.919 ± 0.048	0.688 ± 0.128	0.454 ± 0.049	0.337 ± 0.085

Values are presented as mean ± standard deviation across the five outer validation folds of the nested stratified cross-validation procedure. Clinical models included maternal age, body mass index, history of previous preterm birth, cervical length, and cervical consistency index; metabolomic models included the 30 preselected metabolomic features; and integrated models included both predictor sets. ROC–AUC, area under the receiver operating characteristic curve; PR–AUC, area under the precision–recall curve; PPV, positive predictive value; MCC, Matthews correlation coefficient; XGBoost, Extreme Gradient Boosting; SVM, support vector machine; PLS-DA, partial least squares-discriminant analysis.

## Data Availability

The data that support the findings of this study are available on request from the corresponding author. The computational scripts used for statistical and machine-learning analyses are also available from the corresponding author upon reasonable request.

## Supplementary Materials

The following supporting information can be downloaded at: https://www.mdpi.com/article/10.3390/biomedicines14102188/s1, Figure S1: Confusion matrices for machine-learning models based on clinical data under nested cross-validation; Figure S2: Confusion matrices for machine-learning models based on metabolomic data under nested cross-validation; Figure S3: Confusion matrices for machine-learning models based on integrated clinical and metabolomic data under nested cross-validation; Table S1: Annotation of metabolites detected by untargeted LC-QTOF-MS analysis; Table S2: Additional performance metrics of machine-learning models evaluated using nested stratified cross-validation; Table S3: Pairwise comparison of ROC-AUC across predictor configurations; Table S4: Hyperparameters selected within the inner cross-validation loop across outer folds.

## Abbreviations

The following abbreviations are used in this manuscript: sPTB, spontaneous preterm birth; BMI, body mass index; CCI, cervical consistency index; LC-QTOF-MS, liquid chromatography coupled to quadrupole time-of-flight mass spectrometry; ESI, electrospray ionization; QC, quality control; CV, coefficient of variation; PCA, principal component analysis; PLS-DA, partial least squares-discriminant analysis; ROC, receiver operating characteristic; AUC, area under the receiver operating characteristic curve; ML, machine learning; LR, logistic regression; SVM, support vector machine; DT, decision tree; RF, random forest; XGBoost, extreme gradient boosting; FDR, false discovery rate; VIP, variable importance in projection; TP, true positive; TN, true negative; FP, false positive; FN, false negative; PPV, positive predictive value; NPV, negative predictive value; MSI, Metabolomics Standards Initiative; HMDB, Human Metabolome Database; LPC, lysophosphatidylcholine; LPE, lysophosphatidylethanolamine; PC, phosphatidylcholine; PE, phosphatidylethanolamine; PS, phosphatidylserine; MG, monoacylglycerol.
