# Supplementary material for: Exploratory Integration of Metabolomics and Machine Learning Identifies Candidate Signatures of Spontaneous Preterm Birth
Source: Biomedicines. 2026 Sep 27;14(10):2188. doi: 10.3390/biomedicines14102188 (PMC13701544; doi:10.3390/biomedicines14102188)
Supplement: Supplementary file 1 [file biomedicines-14-02188-s001.zip › biomedicines-4547944-supplementary.pdf]

Supplementary Table S1. Annotation of metabolites detected by untargeted LC-QTOF-MS analysis.

Compound	Molecular formula	Molecular weight	RT (min)	Adduct	Annotation level	Analytical platform	Ionization mode	MS/MS fragments (m/z)	Fold change	p-valor	FDR	SMILES
**Lysophospholipids**												
LPE 16:0	$C_{21}H_{44}NO_7P$	453.2855	**11.62**	M+H	2	LC-QTOF-MS	ESI+	312.2855	1.213463	0.00093194	**0.047037**	[H][C@@](O)(COC(=O)CCCCCCCCCCCCCCC)COP(O)(=O)OCCN
LPC O-14:0	$C_{22}H_{48}NO_6P$	453.3219	**4.13**	M+Na	3	LC-QTOF-MS	ESI+	-	1.472139	0.00020713	**0.023459**	CCCCCCCCCCCCCCOC[C@@H](O)COP([O-])(=O)OCC[N+](C)(C)C
PE P-19:1	C24H48NO6P	477.3219	**11.71**	M+H	2	LC-QTOF-MS	ESI+	184.0733	1.101829	0.001722	0.058245	CCCCCC/C=C\CCCCCCCCC/C=C\OC[C@@H](O)COP(O)(=O)OCCN
LPC 15:0	C23H48NO7P	481.3168	**10.97**	M+H	2	LC-QTOF-MS	ESI+	481.3168, 184.0733	1.162756	0.027232	0.16433	[H][C@@](O)(COC(=O)CCCCCCCCCCCCCC)COP([O-])(=O)OCC[N+](C)(C)C
LPC 17:0	C25H52NO7P	509.6566	**11.71**	M+H	2	LC-QTOF-MS	ESI+	326.6566, 184.0733	1.12174	0.0011122	**0.047047**	CCCCCCCCCCCCC(=O)OC[C@@H](O)COP([O-])(=O)OCC[N+](C)(C)C
LPC 16:0	C24H51NO7P	496.3398	**11.43**	M+H	2	LC-QTOF-MS	ESI+	104.1070, 184.0733, 496.3429	1.104053	0.0053186	0.087587	CCCCCCCCCCCCCCCC(=O)OCC(O)COP(O)(=O)OCC[N+](C)(C)C
LPC 18:1	C26H52NO7P	521.6673	**11.72**	M+H	3	LC-QTOF-MS	ESI+	-	1.034959	0.046541	0.18574	[H][C@@](O)(COC(=O)CCC\C=C/CCCC)COP([O-])(=O)OCC[N+](C)(C)C
LPE 22:6	C27H44NO7P	525.2855	**11.1**	M+H	3	LC-QTOF-MS	ESI+	-	1.138017	0.018655	0.13844	[H][C@@](CO)(COP(O)(=O)OCCN)OC(=O)CC\C=C/C\C=C/C\C=C/C\C=C/C\C=C/C\C=C/CC
**Phospholipids**												
PC 18:1	C26H50NO8P	535.3274	**8.72**	M+H	3	LC-QTOF-MS	ESI+	-	2.642507	0.014027	0.12109	CC(=O)O[C@@H](COP([O-])(=O)OCC[N+](C)(C)C)COC(=O)CCCCCCC/C=C\CCCCCC
PC 18:1 isomer 1	C26H50NO8P	535.3274	**8.72**	M+H	3	LC-QTOF-MS	ESI+	-	2.626424	0.012948	0.11653	CC(=O)O[C@@H](COP([O-])(=O)OCC[N+](C)(C)C)COC(=O)CCCCCCC/C=C\CCCCCC
PC 18:1 isomer 2	C26H50NO8P	535.3274	**8.88**	M+H	3	LC-QTOF-MS	ESI+	-	4.088835	0.029525	0.16433	CC(=O)O[C@@H](COP([O-])(=O)OCC[N+](C)(C)C)COC(=O)CCCCCCC/C=C\CCCCCC
PC 40:5	C48H80NO8P	829.5622	**16.22**	M+H	2	LC-QTOF-MS	ESI+	-	1.202772	0.010587	0.11177	CCCCC\C=C/C\C=C/C\C=C/CCCCCCC(=O)O[C@]([H])(COC(=O)CCC\C=C/C\C=C/C\C=C/C\C=C/C\C=C/CC)COP([O-])(=O)OCC[N+](C)(C)C
PC 38:7 / PE 41:7 / PE-Nme 40:7	C46H78NO8P	803.5465	**15.22**	M+H	3	LC-QTOF-MS	ESI+	-	1.360176	0.0016622	0.058245	
**Phosphatidylserines**												
PS 27:1	C33H62NO10P	663.4111	**13.56**	M+H-H2O	4	LC-QTOF-MS	ESI+	-	2.984435	0.011115	0.11177	CCCCCCCCCCCC(=O)OC[C@@H](COP(O)(=O)OC[C@@H](N)C(O)=O)OC(=O)CCCCCCC/C=C\CCCCC
PS 30:1	C36H68NO10P	705.4581	**14.82**	M+H-H2O	4	LC-QTOF-MS	ESI+	-	2.652526	0.0041078	0.087587	[H][C@](N)(COP(O)(=O)OC[C@@]([H])(COC(=O)CCCCCCC\C=C/CCCC)OC(=O)CCCCCCCCCCCCCCC)C(O)=O
PS 22:0	C28H56NO9P	581.3693	**4.5**	M+H-H2O	4	LC-QTOF-MS	ESI+	-	1.307231	0.029493	0.16433	CCCCCCCCCCCCCCCCCCCCCC(=O)OC[C@@H](O)COP(O)(=O)OC[C@H](N)C(O)=O
**Glycerolipids**												
MG 16:0	C19H38O4	330.277	**14.89**	M+H	3	LC-QTOF-MS	ESI+	-	0.923083	0.015595	0.12447	[H][C@](O)(CO)COC(=O)CCCCCCCCCCCCCCC
**Sphingolipids**												
C17 Sphinganine	C17H37NO2	287.2824	**9.15**	M+H	3	LC-QTOF-MS	ESI+	-	0.564177	0.039752	0.17876	CCCCCCCCCCCCCC[C@@H](O)[C@@H](N)CO
C19 Sphingosine-phosphate	C19H40NO5P	393.2644	**9.5**	M+H-H2O	4	LC-QTOF-MS	ESI+	-	0.718251	0.04693	0.18574	CCCCCCCCCCCCCC/C=C/[C@@H](O)[C@@H](N)COP(O)(O)=O
**Acylcarnitines**												
Myristoleoylcarnitine	C21H40NO4	370.2952	**9.5**	M+Na	3	LC-QTOF-MS	ESI+	-	0.687129	0.044639	0.18574	CCCC\C=C/CCCCCCCC(=O)O[C@H](CC(O)=O)C[N+](C)(C)C
Nonadec-enoylcarnitine	C24H48NO6P	477.3219	**11.43**	M+H	3	LC-QTOF-MS	ESI+	-	1.139653	0.00088043	**0.047037**	CCCCCC/C=C\CCCCCCCCC/C=C\OC[C@@H](O)COP(O)(=O)OCCN
**Lipid aldehydes**												
Hexadecenal	C16H30O	238.2297	**14.89**	M+H	3	LC-QTOF-MS	ESI+	-	0.916248	0.014969	0.12308	CCCCCCCCCCCCC\C=C\C=O
Phenylacetaldehyde	C8H8O	120.0575	**8.96**	M+H	3	LC-QTOF-MS	ESI+	-	1.248273	0.02933	0.16433	O=CCC1=CC=CC=C1
octadecatrienal	C18H30O	262.2297	**15.22**	M+H	3	LC-QTOF-MS	ESI+	-	1.147402	0.031458	0.17007	C(/C/C=C/C/C=C/CC)=CCCCCCCCC([H])=O
**Fatty acids and derivatives**												
DHA	C22H32O2	328.2402	**14.8**	M+H	3	LC-QTOF-MS	ESI+	-	1.157709	0.043423	0.18574	CC\C=C/C\C=C/C\C=C/C\C=C/C\C=C/C\C=C/CCC(O)=O
**Amino acid derivatives**												
Tryptophanol	C10H11NO	161.0841	**11.71**	M+Na	3	LC-QTOF-MS	ESI+	-	1.109783	0.00022183	**0.023459**	OCCC1=CNC2=C1C=CC=C2
**Others**												
Phosphorylcholine	C5H14NO4P	184.0739	**11.43**	M+H	2	LC-QTOF-MS	ESI+	86.0963, 184.0733	1.125597	0.0006561	**0.046255**	C[N+](C)(C)CCOP(O)(O)=O
cholestan pentol disulphate	C27H48O11S2	612.2638	**11.43**	M+H-H2O	4	LC-QTOF-MS	ESI+	-	1.050545	0.03239	0.17038	CC(C)CCC[C@@H](COS(O)(=O)=O)[C@H]1CCC2C3CC[C@@H]4[C@H](O)[C@@H](CC[C@]4(C)[C@H]3[C@@H](O)[C@@H](O)[C@@]21C)OS(O)(=O)=O

Supplementary Table S2. Additional performance metrics of machine-learning models evaluated using nested stratified cross-validation.

Algorithm	Predictor configuration	PR-AUC	Balanced accuracy	Algorithm	Predictor configuration	PR-AUC	Balanced accuracy
**XGBoost**	Clinical	0.382 ± 0.067	0.509 ± 0.056	**SVM**	Clinical	0.468 ± 0.087	0.500 ± 0.000
	Metabolomic	0.646 ± 0.065	0.682 ± 0.050		Metabolomic	0.513 ± 0.126	0.532 ± 0.040
	Integrated	0.630 ± 0.068	0.672 ± 0.051		Integrated	0.617 ± 0.068	0.594 ± 0.041
**Random Forest**	Clinical	0.412 ± 0.055	0.497 ± 0.038	**Decision Tree**	Clinical	0.340 ± 0.038	0.508 ± 0.076
	Metabolomic	0.669 ± 0.106	0.670 ± 0.052		Metabolomic	0.423 ± 0.074	0.583 ± 0.055
	Integrated	0.670 ± 0.083	0.683 ± 0.060		Integrated	0.423 ± 0.058	0.586 ± 0.047
**Logistic Regression**	Clinical	0.461 ± 0.063	0.547 ± 0.061	**PLS-DA**	Clinical	0.452 ± 0.063	0.540 ± 0.024
	Metabolomic	0.601 ± 0.052	0.646 ± 0.070		Metabolomic	0.631 ± 0.063	0.615 ± 0.026
	Integrated	0.655 ± 0.058	0.722 ± 0.053		Integrated	0.642 ± 0.079	0.632 ± 0.031

Supplementary Table S3. Pairwise comparison of ROC-AUC across predictor configurations

Algorithm	Comparison	ΔAUC	95% CI	Unadjusted p-value	FDR-adjusted q-value
**XGBoost**	Integrated vs Clinical	0.248	0.128 to 0.362	<0.001	**0.0009**
	Integrated vs Metabolomic	0.002	−0.054 to 0.059	0.932	0.9324
	Metabolomic vs Clinical	0.246	0.122 to 0.364	<0.001	**0.0009**
**Random Forest**	Integrated vs Clinical	0.241	0.128 to 0.353	<0.001	**0.0009**
	Integrated vs Metabolomic	0.01	−0.008 to 0.029	0.258	0.3096
	Metabolomic vs Clinical	0.231	0.111 to 0.348	<0.001	**0.0014**
**Logistic Regression**	Integrated vs Clinical	0.188	0.076 to 0.298	0.002	**0.0054**
	Integrated vs Metabolomic	0.075	0.006 to 0.150	**0.034***	0.0605
	Metabolomic vs Clinical	0.113	−0.021 to 0.244	0.097	0.125
**SVM**	Integrated vs Clinical	0.227	0.108 to 0.346	<0.001	**0.0009**
	Integrated vs Metabolomic	0.033	−0.035 to 0.103	0.325	0.3654
	Metabolomic vs Clinical	0.194	0.083 to 0.301	0.001	**0.0036**
**Decision Tree**	Integrated vs Clinical	0.118	−0.001 to 0.235	0.052	0.0786
	Integrated vs Metabolomic	0.01	−0.056 to 0.077	0.787	0.8335
	Metabolomic vs Clinical	0.108	−0.010 to 0.225	0.07	0.0964
**PLS-DA**	Integrated vs Clinical	0.195	0.085 to 0.306	0.002	**0.0041**
	Integrated vs Metabolomic	0.025	0.000 to 0.052	0.052	0.0786
	Metabolomic vs Clinical	0.17	0.041 to 0.290	0.008	**0.0168**

Note: Pairwise differences in ROC-AUC (ΔAUC) were estimated using participant-level paired out-of fold predictions obtained from nested stratified cross-validation. Positive ΔAUC values indicate higher discrimination for the first predictor configuration listed in each comparison. Uncertainty was estimated using 5,000 paired stratified bootstrap resamples. P-values were adjusted across the 18 pairwise comparisons using the Benjamini-Hochberg false discovery rate (FDR) procedure. FDR adjusted q-values < 0.05 are shown in bold

Supplementary Figure S1. Confusion matrices for machine-learning models based on clinical data under nested cross-validation. Confusion matrices were constructed from aggregated out-of-fold (OOF) predictions obtained across the five outer validation folds for decision tree (DT), support vector machine (SVM), random forest (RF), Extreme Gradient Boosting (XGBoost), logistic regression (LR), and partial least squares-discriminant analysis (PLS-DA). The clinical-only predictor set comprised maternal age, body mass index, history of previous preterm birth, cervical length, and cervical consistency index (CCI). Each participant contributed one OOF classification generated while excluded from the corresponding outer training set. Binary classifications were obtained using a prespecified threshold of 0.50; for PLS-DA, the cutoff of 0.50 was applied to the continuous PLS response score.

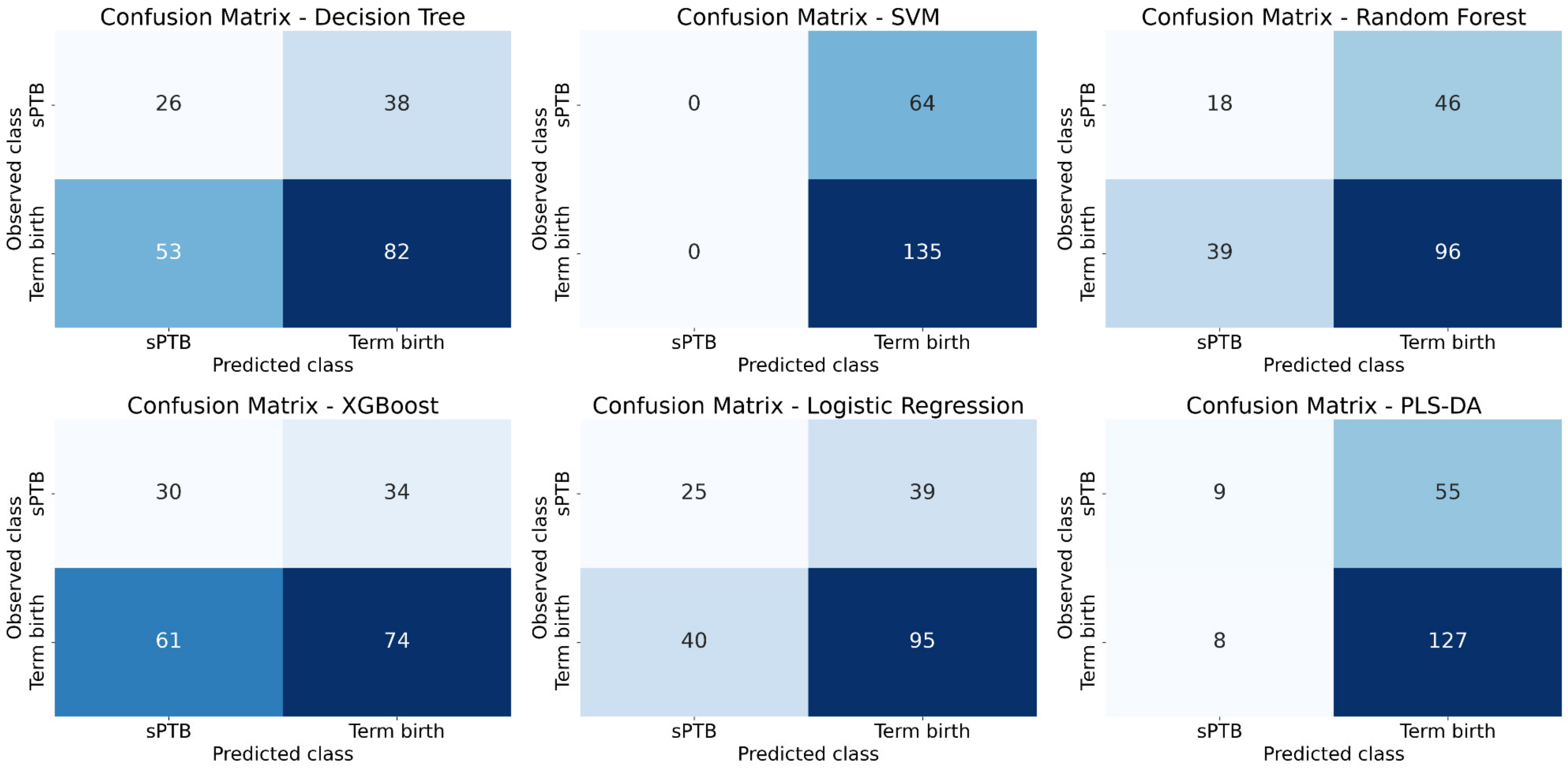

Supplementary Figure S2. Confusion matrices for machine-learning models based on metabolomic data under nested cross-validation. Confusion matrices were constructed from aggregated out-of-fold (OOF) predictions obtained across the five outer validation folds for decision tree (DT), support vector machine (SVM), random forest (RF), Extreme Gradient Boosting (XGBoost), logistic regression (LR), and partial least squares-discriminant analysis (PLS-DA). The metabolomics-only predictor set comprised the 30 preselected metabolomic features. Each participant contributed one OOF classification generated while excluded from the corresponding outer training set. Binary classifications were obtained using a prespecified threshold of 0.50; for PLS-DA, the cutoff of 0.50 was applied to the continuous PLS response score.

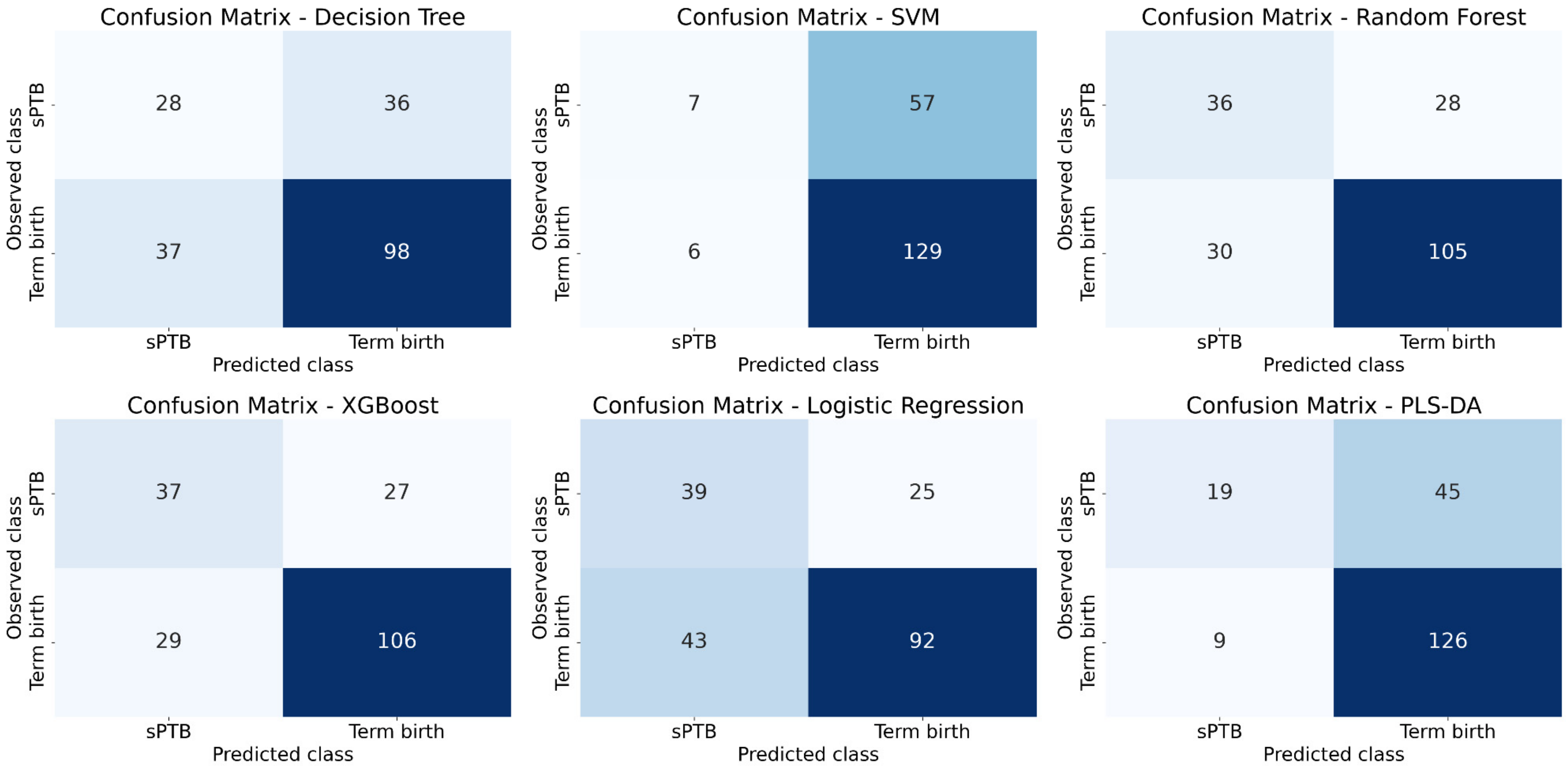

Supplementary Figure S3. Confusion matrices for machine-learning models based on integrated clinical and metabolomic data under nested cross-validation. Confusion matrices were constructed from aggregated out-of-fold (OOF) predictions obtained across the five outer validation folds for decision tree (DT), support vector machine (SVM), random forest (RF), Extreme Gradient Boosting (XGBoost), logistic regression (LR), and partial least squares-discriminant analysis (PLS-DA). The combined predictor set comprised the 30 preselected metabolomic features together with maternal age, body mass index, history of previous preterm birth, cervical length, and cervical consistency index (CCI). Each participant contributed one OOF classification generated while excluded from the corresponding outer training set. Binary classifications were obtained using a prespecified threshold of 0.50; for PLS-DA, the cutoff of 0.50 was applied to the continuous PLS response score.

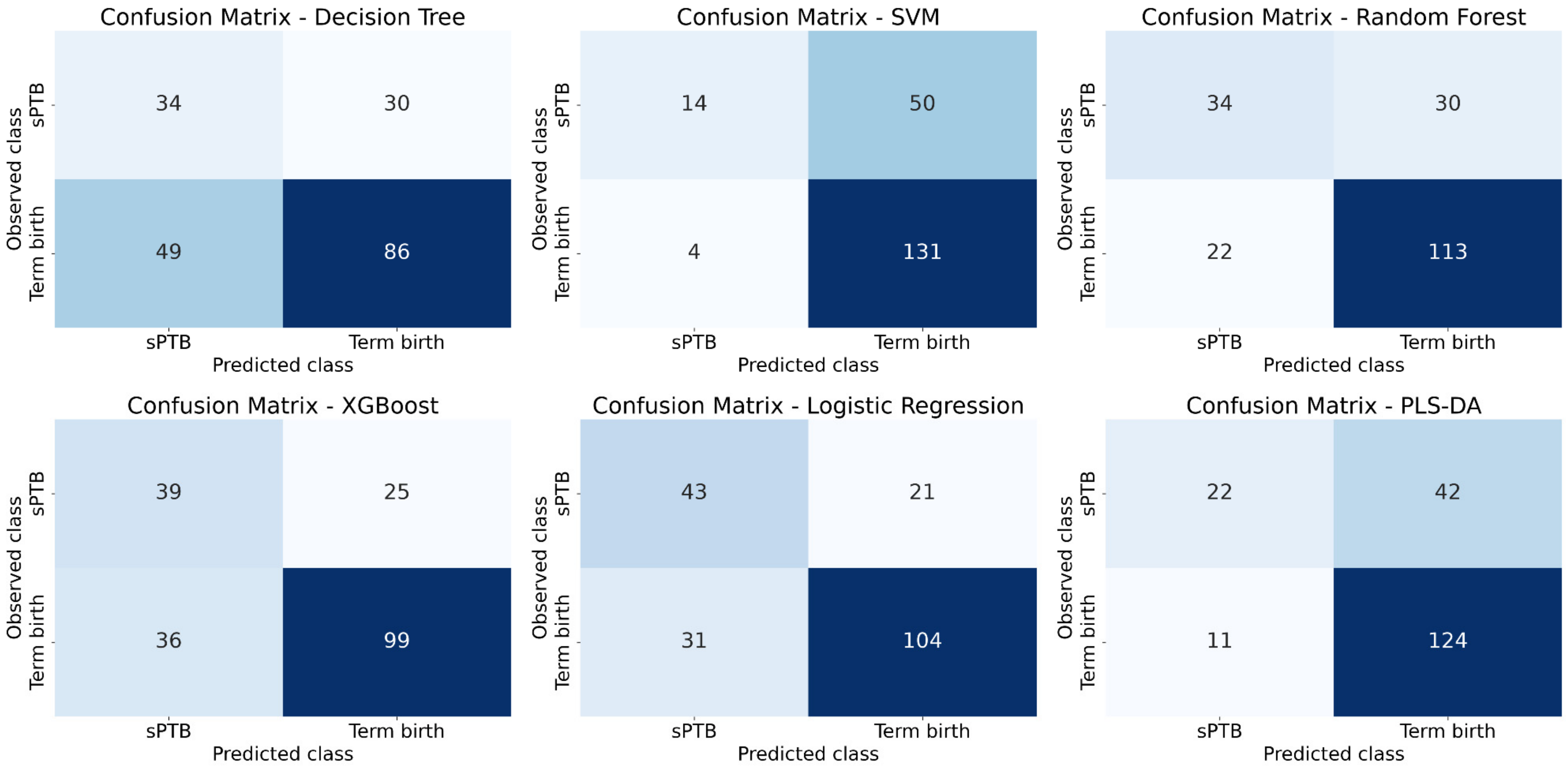

Supplementary Table S4. Hyperparameters selected within the inner cross-validation loop across outer folds

**Predictor configuration**	**Algorithm**	**Outer fold 1**	**Outer fold 2**	**Outer fold 3**	**Outer fold 4**	**Outer fold 5**
Clinical-only	Decision Tree	min_samples_split=5; min_samples_leaf=4; max_features=log2; max_depth=2; criterion=log_loss; ccp_alpha=0; KNN n_neighbors=3	min_samples_split=2; min_samples_leaf=2; max_features=None; max_depth=4; criterion=entropy; ccp_alpha=0.005; KNN n_neighbors=7	min_samples_split=2; min_samples_leaf=2; max_features=sqrt; max_depth=3; criterion=gini; ccp_alpha=0.01; KNN n_neighbors=7	min_samples_split=10; min_samples_leaf=2; max_features=log2; max_depth=3; criterion=gini; ccp_alpha=0; KNN n_neighbors=5	min_samples_split=15; min_samples_leaf=4; max_features=None; max_depth=None; criterion=gini; ccp_alpha=0.005; KNN n_neighbors=7
Clinical-only	SVM	KNN n_neighbors=7; C=0.00409492; kernel=rbf; gamma=1; calibration=sigmoid; calibration CV=5	KNN n_neighbors=5; C=0.494171; kernel=rbf; gamma=0.001; calibration=sigmoid; calibration CV=5	KNN n_neighbors=7; C=6.25055; kernel=linear; gamma=1; calibration=sigmoid; calibration CV=5	KNN n_neighbors=3; C=0.00409492; kernel=linear; gamma=1; calibration=sigmoid; calibration CV=5	KNN n_neighbors=7; C=0.00175751; kernel=rbf; gamma=scale; calibration=sigmoid; calibration CV=5
Clinical-only	Random Forest	n_estimators=600; min_samples_split=10; min_samples_leaf=1; max_features=sqrt; max_depth=7; criterion=gini; bootstrap=True; KNN n_neighbors=7	n_estimators=800; min_samples_split=10; min_samples_leaf=1; max_features=log2; max_depth=3; criterion=entropy; bootstrap=True; KNN n_neighbors=7	n_estimators=200; min_samples_split=10; min_samples_leaf=4; max_features=sqrt; max_depth=3; criterion=entropy; bootstrap=True; KNN n_neighbors=3	n_estimators=400; min_samples_split=2; min_samples_leaf=6; max_features=log2; max_depth=3; criterion=gini; bootstrap=True; KNN n_neighbors=7	n_estimators=600; min_samples_split=10; min_samples_leaf=2; max_features=None; max_depth=10; criterion=entropy; bootstrap=True; KNN n_neighbors=7

Clinical-only	XGBoost	subsample=0.7; reg_lambda=1; reg_alpha=0.01; n_estimators=400; min_child_weight=5; max_depth=5; learning_rate=0.1; gamma=0; colsample_bytree=0.6; KNN n_neighbors=7	subsample=0.7; reg_lambda=1; reg_alpha=0; n_estimators=200; min_child_weight=3; max_depth=2; learning_rate=0.01; gamma=0; colsample_bytree=0.6; KNN n_neighbors=7	subsample=1; reg_lambda=5; reg_alpha=0; n_estimators=100; min_child_weight=1; max_depth=2; learning_rate=0.01; gamma=0; colsample_bytree=1; KNN n_neighbors=7	subsample=1; reg_lambda=5; reg_alpha=0.01; n_estimators=100; min_child_weight=3; max_depth=3; learning_rate=0.01; gamma=0.5; colsample_bytree=0.6; KNN n_neighbors=3	subsample=1; reg_lambda=1; reg_alpha=0.01; n_estimators=100; min_child_weight=3; max_depth=5; learning_rate=0.05; gamma=0.5; colsample_bytree=1; KNN n_neighbors=7
Clinical-only	Logistic Regression	solver=liblinear; C=37.2759; KNN n_neighbors=3	solver=lbfgs; C=0.0372759; KNN n_neighbors=7	solver=lbfgs; C=0.000719686; KNN n_neighbors=5	solver=lbfgs; C=0.00019307; KNN n_neighbors=3	solver=lbfgs; C=1; KNN n_neighbors=5
Clinical-only	PLS-DA	n_components=2; KNN n_neighbors=5	n_components=2; KNN n_neighbors=3	n_components=2; KNN n_neighbors=7	n_components=2; KNN n_neighbors=3	n_components=2; KNN n_neighbors=3
Metabolomics-only	Decision Tree	min_samples_split=10; min_samples_leaf=1; max_features=log2; max_depth=2; criterion=log_loss; ccp_alpha=0.01; KNN n_neighbors=5	min_samples_split=2; min_samples_leaf=2; max_features=None; max_depth=4; criterion=entropy; ccp_alpha=0.005; KNN n_neighbors=7	min_samples_split=15; min_samples_leaf=4; max_features=None; max_depth=None; criterion=gini; ccp_alpha=0.005; KNN n_neighbors=7	min_samples_split=2; min_samples_leaf=2; max_features=None; max_depth=4; criterion=entropy; ccp_alpha=0.005; KNN n_neighbors=7	min_samples_split=10; min_samples_leaf=6; max_features=sqrt; max_depth=None; criterion=gini; ccp_alpha=0.001; KNN n_neighbors=5
Metabolomics-only	SVM	KNN n_neighbors=3; C=1.52642; kernel=linear; gamma=1;	KNN n_neighbors=5; C=0.00308884; kernel=linear; gamma=auto;	KNN n_neighbors=7; C=8.28643; kernel=rbf; gamma=0.01;	KNN n_neighbors=7; C=0.00409492; kernel=rbf; gamma=1;	KNN n_neighbors=7; C=6.25055; kernel=rbf; gamma=scale;

		calibration=sigmoid; calibration CV=5	calibration=sigmoid; calibration CV=5	calibration=sigmoid; calibration CV=5	calibration=sigmoid; calibration CV=5	calibration=sigmoid; calibration CV=5
Metabolomics-only	Random Forest	n_estimators=800; min_samples_split=10; min_samples_leaf=1; max_features=log2; max_depth=3; criterion=entropy; bootstrap=True; KNN n_neighbors=7	n_estimators=800; min_samples_split=10; min_samples_leaf=1; max_features=log2; max_depth=3; criterion=entropy; bootstrap=True; KNN n_neighbors=7	n_estimators=800; min_samples_split=2; min_samples_leaf=1; max_features=0.5; max_depth=3; criterion=entropy; bootstrap=True; KNN n_neighbors=5	n_estimators=800; min_samples_split=10; min_samples_leaf=1; max_features=log2; max_depth=3; criterion=entropy; bootstrap=True; KNN n_neighbors=7	n_estimators=800; min_samples_split=2; min_samples_leaf=1; max_features=0.5; max_depth=3; criterion=entropy; bootstrap=True; KNN n_neighbors=5
Metabolomics-only	XGBoost	subsample=0.85; reg_lambda=1; reg_alpha=0; n_estimators=200; min_child_weight=1; max_depth=3; learning_rate=0.01; gamma=0.5; colsample_bytree=0.6; KNN n_neighbors=5	subsample=0.85; reg_lambda=5; reg_alpha=0.01; n_estimators=400; min_child_weight=5; max_depth=3; learning_rate=0.1; gamma=0; colsample_bytree=0.6; KNN n_neighbors=7	subsample=1; reg_lambda=1; reg_alpha=0.1; n_estimators=200; min_child_weight=1; max_depth=3; learning_rate=0.01; gamma=0; colsample_bytree=0.6; KNN n_neighbors=5	subsample=0.7; reg_lambda=1; reg_alpha=0.01; n_estimators=100; min_child_weight=3; max_depth=2; learning_rate=0.01; gamma=0.5; colsample_bytree=0.6; KNN n_neighbors=7	subsample=0.7; reg_lambda=1; reg_alpha=0.01; n_estimators=100; min_child_weight=1; max_depth=3; learning_rate=0.05; gamma=0; colsample_bytree=1; KNN n_neighbors=7
Metabolomics-only	Logistic Regression	solver=liblinear; C=37.2759; KNN n_neighbors=3	solver=lbfgs; C=0.0026827; KNN n_neighbors=7	solver=liblinear; C=0.00719686; KNN n_neighbors=7	solver=lbfgs; C=0.000517947; KNN n_neighbors=7	solver=lbfgs; C=0.0372759; KNN n_neighbors=7
Metabolomics-only	PLS-DA	n_components=2; KNN n_neighbors=3	n_components=2; KNN n_neighbors=5	n_components=2; KNN n_neighbors=3	n_components=2; KNN n_neighbors=3	n_components=2; KNN n_neighbors=3

Combined	Decision Tree	min_samples_split=15;<br>min_samples_leaf=1;<br>max_features=sqrt;<br>max_depth=5;<br>criterion=entropy;<br>ccp_alpha=0.005;<br>KNN<br>n_neighbors=7	min_samples_split=2;<br>min_samples_leaf=8;<br>max_features=log2;<br>max_depth=3;<br>criterion=log_loss;<br>ccp_alpha=0.005;<br>KNN<br>n_neighbors=7	min_samples_split=15;<br>min_samples_leaf=4;<br>max_features=None; max_depth=None;<br>criterion=gini;<br>ccp_alpha=0.005;<br>KNN<br>n_neighbors=7	min_samples_split=2;<br>min_samples_leaf=2;<br>max_features=None; max_depth=4;<br>criterion=entropy;<br>ccp_alpha=0.005;<br>KNN<br>n_neighbors=7	min_samples_split=5;<br>min_samples_leaf=4;<br>max_features=log2;<br>max_depth=3;<br>criterion=entropy;<br>ccp_alpha=0.005;<br>KNN<br>n_neighbors=5
Combined	SVM	KNN<br>n_neighbors=3;<br>C=1.52642;<br>kernel=linear;<br>gamma=1;<br>calibration=sigmoid; calibration CV=5	KNN<br>n_neighbors=3;<br>C=0.00409492;<br>kernel=linear;<br>gamma=1;<br>calibration=sigmoid; calibration CV=5	KNN<br>n_neighbors=3;<br>C=0.0390694;<br>kernel=linear;<br>gamma=0.01;<br>calibration=sigmoid; calibration CV=5	KNN<br>n_neighbors=5;<br>C=44.9843;<br>kernel=rbf;<br>gamma=0.001;<br>calibration=sigmoid; calibration CV=5	KNN<br>n_neighbors=3;<br>C=0.0390694;<br>kernel=linear;<br>gamma=0.01;<br>calibration=sigmoid; calibration CV=5
Combined	Random Forest	n_estimators=200;<br>min_samples_split=10;<br>min_samples_leaf=2;<br>max_features=sqrt;<br>max_depth=3;<br>criterion=entropy;<br>bootstrap=True;<br>KNN<br>n_neighbors=5	n_estimators=800;<br>min_samples_split=10;<br>min_samples_leaf=1;<br>max_features=log2;<br>max_depth=3;<br>criterion=entropy;<br>bootstrap=True;<br>KNN<br>n_neighbors=7	n_estimators=800;<br>min_samples_split=2;<br>min_samples_leaf=1;<br>max_features=0.5;<br>max_depth=3;<br>criterion=entropy;<br>bootstrap=True;<br>KNN<br>n_neighbors=5	n_estimators=800;<br>min_samples_split=10;<br>min_samples_leaf=1;<br>max_features=log2;<br>max_depth=3;<br>criterion=entropy;<br>bootstrap=True;<br>KNN<br>n_neighbors=7	n_estimators=400;<br>min_samples_split=5;<br>min_samples_leaf=1;<br>max_features=None; max_depth=7;<br>criterion=entropy;<br>bootstrap=True;<br>KNN<br>n_neighbors=3
Combined	XGBoost	subsample=0.7;<br>reg_lambda=1;<br>reg_alpha=0;<br>n_estimators=200;<br>min_child_weight=3; max_depth=2;	subsample=0.7;<br>reg_lambda=1;<br>reg_alpha=0;<br>n_estimators=200;<br>min_child_weight=3; max_depth=2;	subsample=1;<br>reg_lambda=1;<br>reg_alpha=0.01;<br>n_estimators=400;<br>min_child_weight=1; max_depth=2;	subsample=1;<br>reg_lambda=1;<br>reg_alpha=0.1;<br>n_estimators=200;<br>min_child_weight=3; max_depth=3;	subsample=1;<br>reg_lambda=1;<br>reg_alpha=0;<br>n_estimators=400;<br>min_child_weight=3; max_depth=2;

		learning_rate=0.01; gamma=0; colsample_bytree= 0.6; KNN n_neighbors=7	learning_rate=0.01; gamma=0; colsample_bytree= 0.6; KNN n_neighbors=7	learning_rate=0.1; gamma=0.5; colsample_bytree= 0.8; KNN n_neighbors=3	learning_rate=0.01; gamma=0.5; colsample_bytree= 0.8; KNN n_neighbors=7	learning_rate=0.01; gamma=0.5; colsample_bytree= 0.8; KNN n_neighbors=7
Combined	Logistic Regression	solver=lbfgs; C=1; KNN n_neighbors=5	solver=lbfgs; C=0.000719686; KNN n_neighbors=5	solver=lbfgs; C=0.0372759; KNN n_neighbors=7	solver=liblinear; C=0.1; KNN n_neighbors=3	solver=lbfgs; C=0.13895; KNN n_neighbors=3
Combined	PLS-DA	n_components=2; KNN n_neighbors=3	n_components=2; KNN n_neighbors=3	n_components=2; KNN n_neighbors=3	n_components=2; KNN n_neighbors=3	n_components=2; KNN n_neighbors=3

Note: Hyperparameters were optimized independently within the 5-fold inner cross-validation of each outer-training fold. Therefore, selected values may differ across outer folds. For PLS-DA, n_components = 2 was prespecified and was not optimized according to outcome performance; KNN n_neighbors was selected within the cross-validation procedure. SVM calibration used sigmoid calibration with 5-fold calibration restricted to the outer-training data. Inner-CV AUC values are not shown because they are tuning results rather than hyperparameters.
